# Brain Structural and Functional Connectivity: A Review of Combined Works of Diffusion Magnetic Resonance Imaging and Electro-Encephalography

**DOI:** 10.3389/fnhum.2021.721206

**Published:** 2021-10-07

**Authors:** Parinaz Babaeeghazvini, Laura M. Rueda-Delgado, Jolien Gooijers, Stephan P. Swinnen, Andreas Daffertshofer

**Affiliations:** ^1^Department of Human Movements Sciences, Faculty of Behavioural and Movement Sciences, Amsterdam Movement Science Institute (AMS), Vrije Universiteit Amsterdam, Amsterdam, Netherlands; ^2^Institute for Brain and Behaviour Amsterdam (iBBA), Faculty of Behavioural and Movement Sciences, Vrije Universiteit, Vrije Universiteit Amsterdam, Amsterdam, Netherlands; ^3^Movement Control & Neuroplasticity Research Group, Department of Movement Sciences, KU Leuven, Leuven, Belgium; ^4^Trinity Centre for Biomedical Engineering, Trinity College Dublin, The University of Dublin, Dublin, Ireland; ^5^KU Leuven Brain Institute (LBI), Leuven, Belgium

**Keywords:** diffusion-based magnetic resonance imaging (dMRI), electro-encephalography (EEG), white matter (WM) microstructural organization, functional connectivity, event-related potentials (ERPs), resting state, spectral analysis

## Abstract

Implications of structural connections within and between brain regions for their functional counterpart are timely points of discussion. White matter microstructural organization and functional activity can be assessed in unison. At first glance, however, the corresponding findings appear variable, both in the healthy brain and in numerous neuro-pathologies. To identify consistent associations between structural and functional connectivity and possible impacts for the clinic, we reviewed the literature of combined recordings of electro-encephalography (EEG) and diffusion-based magnetic resonance imaging (MRI). It appears that the strength of event-related EEG activity increases with increased integrity of structural connectivity, while latency drops. This agrees with a simple mechanistic perspective: the nature of microstructural white matter influences the transfer of activity. The EEG, however, is often assessed for its spectral content. Spectral power shows associations with structural connectivity that can be negative or positive often dependent on the frequencies under study. Functional connectivity shows even more variations, which are difficult to rank. This might be caused by the diversity of paradigms being investigated, from sleep and resting state to cognitive and motor tasks, from healthy participants to patients. More challenging, though, is the potential dependency of findings on the kind of analysis applied. While this does not diminish the principal capacity of EEG and diffusion-based MRI co-registration, it highlights the urgency to standardize especially EEG analysis.

## Introduction

The human brain is characterized by structural and functional connectivity within and between regions. Structural connectivity refers to the anatomical organization of the brain by means of fiber tracts. Recent advances in magnetic resonance imaging (MRI) and image processing provide various means to quantify structural connectivity in a non-invasive way using short-range local measures and/or long-range tract tracing procedures, called diffusion tractography. Functional connectivity refers to statistical dependence between time series of electro-physiological activity and (de)oxygenated blood levels in distinct regions of the brain. The first can be assessed non-invasively using encephalographic recordings. Electro- and magnetoencephalography (EEG and MEG, respectively) reflect changes in averaged post-synaptic potential and dendritic currents of neural populations and, hence, provide *direct insight* into ‘neural functioning’. With the latter, typically based on functional MRI (fMRI) and blood-oxygen-level dependent (BOLD) contrasts, one assesses (correlated) changes in the metabolic demand, providing *indirect insight* into this neural functioning.

Structural connectivity is known to shape functional connectivity (Honey et al., 2010) but the extent to which it does may vary substantially. To pinpoint this further, one may directly relate structural and functional assessments by co-registering diffusion MRI (dMRI) and EEG or fMRI. Many studies followed this idea and combined dMRI with fMRI, in parts with great success (Mulkern et al., 2006; Johansen-Berg, 2012; Bennett and Rypma, 2013). As mentioned above, however, BOLD signals are indirect measures of neural functioning. They include complex convolutions of not only neural, but also vascular signals (Logothetis, 2008), which may jeopardize the interpretation of fMRI outcomes. This has been shown, e.g., in studies in older adults and patients with cerebrovascular disease, impaired cerebrovascular dynamics and impaired neurovascular coupling (Krainik et al., 2005; Logothetis, 2008; Tsvetanov et al., 2015). Using fMRI in combination with EEG can help circumventing this possible confounder. More important, though, EEG allows for a direct assessment of neural activity, first and foremost in cortical regions. What remains to be shown, however, is whether the combination of EEG and dMRI can really help to identify the relationship between structural and functional connectivity in a consistent manner. With the current review we seek to provide a first inventory on more recent studies employing both recording modalities in unison.

### Electro-Encephalography

**FIGURE 1:**
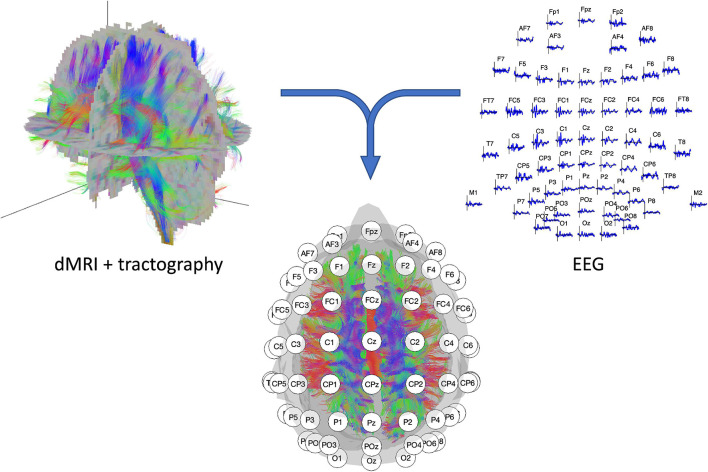
Illustration of the two recording modalities. **(Left top panel)** dMRI recording with resulting fiber tracts. **(Right top panel)** 64-channel EEG signal with electrode layout conform the 10–20 systematic. **(Middle lower panel)** EEG/dMRI combination to explore the link between structural and functional connectivity.

Electro-encephalography signals are recorded from the scalp using surface electrodes at positions typically based on the 10–20 system (Niedermeyer and Lopes da Silva, 1993); see Figure 1, below. They predominantly reflect activity of pyramidal neurons or, more generally, electrical dipoles between soma and apical dendrites generated by summed postsynaptic potentials of large neuronal populations (Niedermeyer and Lopes da Silva, 2004). EEG recordings can serve to monitor ongoing/spontaneous or evoked/induced neural activity. The first typically involves spectral analysis to discern the functional relevance of spectral characteristics in distinct frequency bands (e.g., theta ∼4–8 Hz, alpha ∼8–14 Hz, beta ∼15–30 Hz, or gamma ∼>30 Hz). The latter relies on the study of so-called event-related potentials (ERPs), i.e., signal responses that co-vary with in- or external stimuli. For both cases, various analysis methods are available to estimate neural activity in the brain (∼ source activity) from scalp signals. Be it on electrode or source level, be it ongoing or evoked activity, ERP activity and the spectral power are considered markers for local synchronization in comparably small neural populations, while distant synchronization is defined as statistical interdependence (synchronization or, more general, correlation) between regions-of-interest (ROI).

### Diffusion Magnetic Resonance Imaging

*Diffusion MRI* encodes diffusion-driven displacements of water molecules over time into MRI signals. It allows to assess tissue characteristics *in vivo* (Stejskal and Tanner, 1965). In the presence of gradient pulses, diffusion attenuates the MRI signal rendering it diffusion weighted. In contrast to freely diffusing water, biological tissue hinders and/or restricts molecular diffusion, which can provide information about the corresponding microstructural organization. Diffusion tensor imaging (DTI) (Basser, 1995) is a widely used dMRI analysis approach to obtain anatomic features of white matter (WM). However, DTI is based on the assumption that the molecular displacement distribution in biological tissue is Gaussian (Basser, 1995), while in reality it is distinctly non-Gaussian (Assaf et al., 2004). The degree of (symmetric) deviation from the Gaussian distribution can be quantified via the kurtosis. Diffusion kurtosis imaging (Jensen and Helpern, 2010) exploits this via so-called multi-shell high angular resolution diffusion imaging. It can provide additional and often relevant information about tissue heterogeneity.

The dMRI comes with several outcome measures, all meant to quantify the WM microstructural organization. The mostly used metrics are fractional anisotropy (FA), three forms of diffusivity, and kurtosis anisotropy (KA). FA quantifies the amount of diffusion along the principal direction on a normalized scale, i.e., a value of 0 indicates undirected or isotropic diffusion, whereas a value of 1 indicates full directedness or anisotropy. FA is thought to reflect various tissue properties such as axonal myelination, axonal diameter, axonal density, and fiber organization (Scholz et al., 2009). By contrast, diffusivity measures quantify the overall degree of water diffusion. As said, it is specified in three forms: mean diffusivity (MD), radial diffusivity (RD), and axial diffusivity (AD), all in relation to the aforementioned principal direction of diffusion (Pierpaoli and Basser, 1996). Lastly, KA solely reflects the anisotropy of the kurtosis tensor without contributions from the diffusion tensor (Poot et al., 2010). All the measures can be estimated in isolated ROIs or for fibers connecting them, the latter after conducting additional tractography; see Figure 1.

### A Mechanistic View

From a *mechanistic perspective* one may expect that the nature of WM microstructural organization influences the transfer of activity between different neuronal populations. That is, if EEG measures serve to quantify such transfer of activity directly, one may expect them to be strongly associated with the afore-listed DWI metrics. By hypothesis, a higher degree of microstructural organization is, hence, accompanied by stronger and faster neural transmission. As will be shown, this is indeed the case for some of the EEG outcomes but not for all of them.

## Approach and Outline

### Literature Review

We conducted a keyword-based search in PubMed and ISI Web-of-Science. Search terms [“DTI” AND “EEG”], [“DWI” AND “EEG”], and [“DKI” AND “EEG”], which provided 270, 220, and 3 hits, respectively. Animal and computational studies, technical/methods studies as well as clinical studies that employed the modalities for their isolated diagnostic value were excluded. We also excluded review papers and non-English publications. This selection yielded 40 combined dMRI/EEG studies including 17 experimental ones in healthy participants and 23 investigating pathology. The latter included neurodegenerative disease like epilepsy and Alzheimer, trauma-induced cases like stroke and traumatic brain injury (TBI), and psychiatric conditions (e.g., schizophrenia); an overview of some of them can be found as Supplementary Material.

Estimating the relationship between structural and functional brain characteristics requires three important steps: (*i*) selecting a set of ROIs, either *a priori* or based on the statistical significance of FA group differences or signal power as a function of location; (*ii*) defining appropriate (statistical) methods to determine the structural and functional characteristics of interest; and (*iii*) selecting the proper statistical means to identify possible associations between them. Several studies (*N* = 13) refrained from conducting step (*iii*) – for example because they referred to case studies – and only reported the individual EEG- and dMRI-based results. Yet, these studies revealed novel insight into the structure/function relationship and were included in this review.

### Outline

We arranged our inventory of studies by experimental paradigm and corresponding analysis method, as both factors turned out to qualitatively affect the outcomes regarding the (expected) relation between functional EEG and structural dMRI measures. Given the long tradition of using EEG both in fundamental research and in the clinic, its experimental designs and analyses form the backbone of our review, from assessments of event-related potentials, different spectral analyses and functional connectivity approaches to more recent studies involving source-localization techniques. In what follows we will particularly highlight the reported association between these functional activities and co-registered dMRI measures before discussing the potential benefits and shortcomings of such combined recordings.

## Event-Related Potentials

The most used approach in experimental EEG studies comprises of evoking activity patterns using repeated stimuli that may differ between studies. This design yields so-called event-related potentials (ERPs), highlights activity that is time-locked to an event (i.e., the moment of stimulation) and eliminates non-time-locked background activity via averaging to event-aligned epochs. According to the aforementioned mechanistic view (see Section “A Mechanistic View”), the possibility of time-locked activity to emerge depends on the myelination of connecting axons and dendrites (Pajevic et al., 2013; Noori et al., 2020). An increased degree of microstructural organization correlates with faster action potential conduction (Westerhausen et al., 2006; Whitford et al., 2011a). Especially FA is believed to reflect this degree of WM microstructure and should therefore be associated with different outcome measures like ERP amplitudes and latencies.

All the reports included in this section are so-called *task-related studies*, where participants were asked to perform a cognitive, sensory, or motor task during EEG recording. We grouped them under the headings *error-related*, *early attention-related* and *later cognitive-related responses*. Error-related responses are ERPs evoked by commission errors when participants are asked to respond as accurately and quickly as possible to a cognitive task (Gehring et al., 1993). Early attention-related responses can be observed when the task is to react (respond) as soon as an external sensory stimulus has been observed (Noesselt et al., 2002; Poghosyan et al., 2005). Cognition-related responses are later ERPs resulting from attention and cognitive control processes, e.g., when participants respond to stimuli like words or images (Patel and Azzam, 2005).

### Error-Related Responses

Westlye et al. (2009) studied the interaction between error-related negativity (ERN) and WM characteristics during a (modified) Eriksen flanker task (Eriksen and Eriksen, 1974). The ERN originates from the cingulate cortex (Herrmann et al., 2004; Debener et al., 2005) and has a negative peak ∼40–70 ms after a behavioral/cognitive error has been committed (Gehring et al., 1993; Taylor et al., 2007). Westlye et al. (2009) found a positive correlation between ERN amplitudes over medial fronto-central EEG electrodes and mean FA values in left posterior cingulate cortex and left superior longitudinal fasciculus. They also reported a negative correlation between the ERN amplitude size and RD values, i.e., smaller RD values correlated with more negative ERN amplitudes in the left cingulum bundle. According to Westlye et al. (2009) the WM microstructural organization in anterior cingulate cortex predicts amplitude of the ERN component though strictly speaking they did not provide a corresponding proof for this conjecture. Gao et al. (2017) followed a similar paradigm using the Eriksen flanker task but they focused on the N200 (or N2) amplitudes as a measure of response conflict (Clayson and Larson, 2013). N2 amplitudes were determined as maximum negativity averaged over electrodes (Yeung et al., 2004) and FA maps were estimated from a whole-brain voxel-wise analysis using tract-based spatial statistics (TBSS), which refers to the projection onto an alignment-invariant tract representation (the “mean FA skeleton”) after a properly tuned non-linear registration of individual FA images (Smith et al., 2006). With this, Gao et al. (2017) found a significant positive correlation between larger N2 amplitudes and higher FA in right superior longitudinal fasciculus connecting frontal and parietal cortex. This suggests a direct relationship between WM-based structural connectivity and EEG measures of cognitive responses.

### Attention-Related, Early Responses

Myer et al. (2016) evaluated the so-called Q-Collar, a neck collar worn by athletes with the intention to protect the brain from head impacts. Aim of this study was to test the collar’s effect on reducing neuroanatomical and neurophysiological damage in two groups of hockey players that either wore the collar or did not (the latter served as controls). DTI was collected at different time points (pre- and mid-season) at which also EEG data were collected while the athletes performed an auditory oddball paradigm. Latencies and amplitudes of the ERPs served to compute so-called brain network activation (BNA) scores. As compared to the collar group, controls showed larger electrophysiological changes along with increased MD/RD values from pre- to mid-season in the corpus callosum. Only in the control group, significant positive correlations were found between an increase in the absolute change of BNA scores and increased MD/RD values in various ROIs. It seems that structural alterations yield larger ERP latencies and lower ERP amplitudes.

Lateralized visual stimuli allow for unravelling the interplay between left and right hemispheres. Westerhausen et al. (2006) followed this idea and investigated both reaction time- and evoked potential-based inter-hemispheric transmission times (IHTTs) and their relation to corpus callosum architecture. Healthy participants responded to briefly presented lateralized visual stimuli. IHTT can be considered a measure of interhemispheric, functional connectivity. IHTT was estimated from P100 and N160 ERP components in lateral electrode pairs over occipital, parietal, and temporal cortices. Given the focus on the corpus callosum, DWI measures were extracted for the genu, truncus, and posterior third. This revealed significant correlations between longer left/right occipital P100 IHTT and lower MD values in callosal regions connecting visual cortical areas. While this seemingly contradicts the aforementioned mechanistic account, it does suggest a direct relationship between microstructural organization of the posterior regions of the corpus callosum and inter-hemispheric transfer time of visual information.

To further detail spatial dependencies, Whitford et al. (2011a) applied tractography between selected ROIs. They investigated the correlation between IHTT and structural connectivity but concentrated on fibers of the corpus callosum connecting visual processing areas in groups of schizophrenia patients. Unilateral visual stimuli were presented, and participants were asked to look at a central fixation cross and count the number of targets. IHTT was estimated for N100 and P100 (or P1) components, which were determined as the most negative and positive amplitudes post stimulus in the bilateral, parietal electrode pairs. Rather than correlating ERP amplitudes, Whitford et al. (2011a) used the left/right difference in peak latencies and compared them with FA values extracted from visual fibers crossing the posterior part of the corpus callosum, bilaterally connecting primary and secondary visual cortices. They found an association between longer IHTT (P100 latency differences) and lower FA values in visual callosal fibers for both groups, albeit not significant after Bonferroni correction. The relationship was found in fasciculi that were shown to be structurally affected in schizophrenia (Kubicki et al., 2007; Whitford et al., 2011b). Apparently, changes in the WM microstructural organization of the visual fibers of the corpus callosum are accompanied by additional conduction delays in schizophrenia patients. Using a lateralized cognitive task (dichotic listening), Friedrich et al. (2017) searched for correlations between individual differences in neural architecture of the corpus callosum and functional asymmetries, i.e., hemispheric latency differences. They determined the N100 ERP amplitudes in lateral central electrodes, which are believed to relate to bottom-up attentional processes (Herrmann and Knight, 2001). Left/right differences in peak latencies were compared with mean FA values in anterior callosal third, mid segment and posterior callosal third. The results indicated higher FA values to be associated with reduced hemispheric latency differences, as found in the posterior callosal third during the dichotic condition.

Turning to the interaction between the auditory and the motor network, Whitford et al. (2011c) conducted an experimental study in two groups of schizophrenia patients and healthy controls. They employed three experimental conditions to deliver self-generated auditory stimuli with different delays (0, 50, and 100 ms) after a button-press. They also included a condition coined “passive-listening”, in which auditory stimuli were delivered without button presses. From the central, medial electrode, the N1 suppression was determined as the difference between the N1 amplitude during self-generated auditory stimuli and that during the passive-listening condition. This amplitude is known to be suppressed in response to self-generated auditory stimulation (Schafer and Marcus, 1973; McCarthy and Donchin, 1976; Ford et al., 2001, 2007; Martikainen et al., 2005). And, according to Ford et al. (2007), schizophrenia patients exhibit subnormal amounts of N1 suppression to auditory stimulation evoked indirectly by a voluntary motor action. In Whitford et al. (2011c), FA values were estimated for the left arcuate fasciculus tracts, connecting motor initiation areas in the frontal lobe with auditory cortex. They observed a significant positive correlation between the corresponding FA values and the degree of N1 suppression in the un-delayed condition (higher FA values correlated with higher N1 suppression) and a negative correlation with N1 suppression in one of the delayed conditions (50 ms). Although this was the case for both schizophrenia patients and healthy controls, they speculated that structural damage to the arcuate fasciculus may have been responsible for the observed delays in corollary discharges arrival at sensory cortex in schizophrenia patients. This agrees with the idea that corollary discharges are involved in suppressing the sensory consequences of self-generated actions (Crapse and Sommer, 2008).

### Cognition-Related, Later Responses

Evstigneev et al. (2013) assessed the relationship between structural and clinical features in patients with pharmaco-resistant epilepsy, patients in persistent remission and healthy controls during an auditory two-tone oddball paradigm. The P300 component typically occurs after sensory discrimination (Picton, 1992). Evstigneev et al. (2013) extracted this ERP from the side of epileptic activity focus. FA was estimated for anterior and posterior quadrants of axial sections. They found a significant, negative correlation between epileptic activity and FA values, in which increased amplitude of theta rhythm correlated with decreased FA values in the hemisphere with epileptic activity. Evstigneev et al. (2013) also reported that FA values were negatively correlated with a latency period of the P300 in the anterior quadrant of the epileptogenic hemisphere (decreased FA values correlated with a longer P300 latencies). Since the P300 generation seems to involve interhemispheric integration of sensory information (Knight et al., 1989; Yamaguchi and Knight, 1991, 1992), the P300 might be affected by altered transcallosal fibers (Caminiti and Sbriccoli, 1985; Pandya and Seltzer, 1986). The results hence suggest that P300 latencies can be affected by microstructural organization of the corpus callosum. Put differently, the microstructural organization of the corpus callosum seems to correlate with cognitive functions.

Oestreich et al. (2019) investigated whether quantifying structural properties of auditory WM pathways may improve the prediction of psychotic-like experiences in the healthy population over and above predictions made by auditory prediction error responses alone. Participants listened to a classical two-tone duration deviant oddball paradigm and a stochastic oddball paradigm. They were asked to focus on a visual task (stream of letters) and detect consecutive repetitions of presented letters. ERPs were estimated for each condition and mismatch negativity was extracted. Mismatch negativity is a component of prediction errors, evoked by stimuli that differ from a learnt pattern (Belger et al., 2012; Nagai et al., 2013; Näätänen, 2014). Its amplitude has been shown to be reduced in schizophrenia. As indicator of the prodromal stage makes it a candidate biomarker for schizophrenia (Light and Näätänen, 2013; Näätänen et al., 2016). The left/right arcuate fasciculi and auditory interhemispheric tracts were reconstructed (cf. Table 1). Reduced mismatch responses (less-negative amplitudes) and reduced FA of the auditory interhemispheric pathway were associated with psychotic-like experiences. Both of them may hence marker early psychotic symptoms.

**TABLE 1:** Studies on event-related potentials, including positive (+) or negative (−) associations between ERP amplitudes (A) or latencies (L) and DTI metrics.

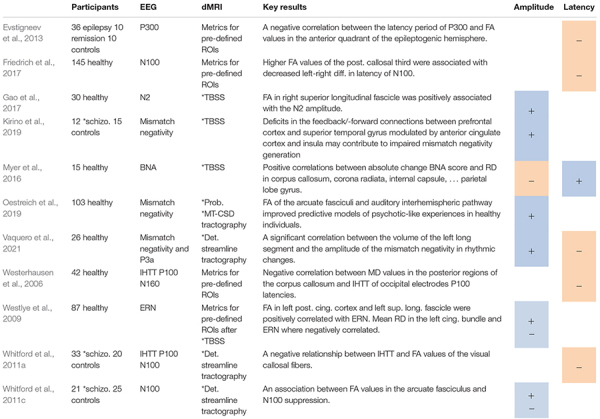

*MT-CSD, multi-tissue constrained spherical deconvolution; TBSS, tract-based spatial statistics; Schizo, schizophrenia; Det, deterministic; Prob, probabilistic.

Kirino et al. (2019) examined the mismatch negativity responses elicited by omission of auditory stimuli and their association with structural abnormalities in anterior cingulate cortex in patients with schizophrenia. Mismatch negativity amplitudes (at frontal and central electrode at midline) were expected to be reduced in patients with schizophrenia (Koshiyama et al., 2018). EEG served primarily a regressor of the co-registered fMRI revealing significant BOLD changes in the left superior temporal gyrus, anterior cingulate cortex, right superior temporal gyrus, and inferior/mid frontal cortices (Oknina et al., 2005). In patients, the amplitudes also correlated positively with BOLD in the Heschl’s gyrus. By contrast, FA values in the left anterior cingulate cortex were positively correlated with BOLD changes in right superior temporal gyrus. Kirino et al. (2019), thus, suggested that deficits of feedback/-forward connections between prefrontal cortex and superior temporal gyrus modulated by the anterior cingulate cortex and insula are related to impaired mismatch negativity generation.

Vaquero et al. (2021) assessed correlations between macrostructural properties of the arcuate fasciculus connecting potential cortical sources of mismatch negativity during music perception. Leading hypothesis was that mismatch negativity and frontal novelty P3 (P3a) are elicited after deviant responses. Mismatch negativity reflects the pre-attentive detection of a difference between an incoming sound and the predicted one (Näätänen et al., 2007; Winkler et al., 2009; Koelsch et al., 2019) and the P3a is associated with a shift in attention toward a surprising sound (Nager et al., 2003; Horváth et al., 2008; Berti, 2013). Tract volume measures were estimated for selected ROIs. A larger volume of the left long segment of the arcuate fasciculus was correlated with larger mismatch negativity amplitudes in a paradigm with rhythmic stimuli. Vaquero et al. (2021) argued that the lateralization of correlation reflects a specialization of left auditory regions in detecting changes in rhythmic stimuli (Zatorre et al., 1992; Zatorre et al., 2002; Poeppel, 2003).

### Conclusion

Event-related potential components can reveal the transmission of signals between neuronal populations. As such they can provide insight into neural information transfer. Effects of structural connectivity on functional activity may explain differences in amplitudes and latencies of ERP components, both in healthy participants and patients (see Table 1). Changes in WM microstructural organization may affect the latency of the ERP component and velocity of data transfer between different brain regions, in particular between hemispheres (Westerhausen et al., 2006; Whitford et al., 2011a; Friedrich et al., 2017). ERP components can hence be considered sensitive to microstructural differences and, in consequence, amplitudes and latencies of ERP components may reflect structural changes in pathology during cognitive, sensory, or motor performance.

## Spectral Analysis – Local Functional Connectivity

In contrast to the ERP studies described in *Section “Event-Related Potentials,”* the studies regarding the spectral composition of (ongoing) activity entails both *resting state* and *task-related* assessments, a classification we adopt to group the here-included ones. The so-called resting state condition refers to EEG recordings during which participants are asked to relax and ‘do nothing’ for some time period (i.e., neither cognitive, sensory nor motor tasks were imposed). Recently, resting state studies have become quite popular but more traditionally the EEG’s spectral content has been associated with task-related functional state like attention or motor performance. Given the diversity of EEG studies supporting the latter, spectral power in distinct frequency bands and its association with structural measures has in fact been the primary target in the search for functional expression of WM microstructural changes (Colrain et al., 2011; Olbrich and Arns, 2013; Scrascia et al., 2014). Hence, we organized the here-reviewed experimental reports by the frequency bands under study.

### Delta and Theta Oscillations

Delta and theta oscillations cover low frequency ranges (∼0.5–4 and ∼4–8 Hz, respectively). They can be observed, e.g., during the deepest level of relaxation or sleep but also mark anxiety, poor emotional awareness, stress, depression, inattentiveness and dementia (Teplan, 2002; McFarlane et al., 2005; McLaughlin et al., 2010; Gemignani et al., 2012; Scrascia et al., 2014; Bernardi et al., 2016).

#### Resting State Studies

Scrascia et al. (2014) investigated the relationship between WM alterations and functional abnormalities from eyes-closed resting state EEG data in older adults with mild cognitive impairment and Alzheimer’s disease and contrasted this with data from age-matched healthy controls. Focus was on the spectral power per EEG electrode. In the left frontal WM and in the corpus callosum, the progressive decrease in FA values appeared to be significantly correlated with increased delta and theta rhythm in frontopolar and frontal ROIs. Scrascia et al. (2014) speculated frontal areas to be the most vulnerable areas in Alzheimer disease that show the ‘abnormal’ functional activity due to the macrostructural atrophy. A comparable eyes-closed resting state paradigm was used by Duru et al. (2016) in patients with thalamic stroke. They found a significant correlation between FA values and delta/theta power metrics (averaged over electrodes) for patients and age-matched healthy controls. In healthy controls, delta power was correlated with FA values in the left superior corona radiata (connecting medial frontal cortex and basal ganglia), which was absent in the patients. The theta power was correlated with FA values in the body of corpus callosum, the left cingulum, and in bilateral superior longitudinal fasciculi in the healthy controls, and in patients in the right external capsule. Unfortunately, Duru et al. (2016) did not report the direction of correlation between FA values and EEG power. However, previous studies found negative correlations between similar parameters in stroke patients (Sainio et al., 1983; Nuwer et al., 1987; Calixto et al., 2004). For this, we speculate that the increased delta and theta power were correlated with decreased FA values.

Bruno et al. (2011) used multimodal imaging to study the unresponsive wakefulness syndrome and minimally conscious state induced by trauma. Since trauma was the reason of unconsciousness in these two patients, the authors explicitly asked whether the observed functional abnormalities were related to axonal or cortical damage. They considered EEG power per hemisphere during an eyes-closed resting state condition and used a left/right asymmetry index. For this, they subtracted the EEG-power of the right hemisphere from that of the left one and divided that difference by the sum of the two. From the DTI data, the number of WM tracts per hemisphere was extracted, in line with asymmetry analysis. Correlations between EEG and DTI outcomes were not determined. Yet, non-reactive delta (4–5 Hz) dysrhythmia with larger amplitude over the left hemisphere appeared to coincide with a marked reduction of the number of WM tracts in the same hemisphere in unresponsive wakefulness syndrome. By contrast, DTI tracts appeared symmetric in the minimally conscious state. These results suggest that unconsciousness in the unresponsive wakefulness syndrome is not only due to altered functional connectivity between brain areas but is accompanied by structural damage. For the minimally conscious state this is not necessarily the case.

#### Task-Related Studies

Colrain et al. (2011) evaluated the effect of alcoholism in older participants on the time-resolved spectral power and on the WM microstructural organization. They were particularly interested in whether (earlier reported) changes in spectral power correlate with structural changes. Participants conducted a reaction time task for a visual Go/No-Go protocol representing an “oddball” Bernoulli series of letters. Relative to healthy, age-matched controls, alcohol addicts exhibited significantly lower delta power during the No-Go condition, which correlated with increased RD values in left and right cingulate bundles. In consequence, reduced delta power might be considered an electrophysiological expression for WM degradation in the cingulate bundle linking frontal to parietal cortical regions.

#### Sleep Studies

Given the close relation between sleep, cognitive performance and memory consolidation, Bernardi et al. (2016) examined the relationship between sleep parameters and brain structural changes after 12 and 24 h of intensive task practice and after post-training sleep (recovery sleep) in healthy participants. Spectral power was averaged over EEG electrodes during non-REM sleep and the delta power and its changes between the first and the last non-REM sleep cycle were considered during recovery sleep. Participants performed a driving simulation game, or a set of tasks based on impulse control, decision-making and conflict resolution. The learning paradigm was chosen because learning may lead to swift microstructural changes in gray matter (GM) and WM. MD values dropped significantly in cortical GM after intense training when sleep deprived but reverted after recovery sleep. The authors could not establish significant associations between changes in MD values (in both GM and WM) after recovery sleep and differences of delta power between the first and the last non-REM sleep cycles.

Gemignani et al. (2012) studied changes in power in various frequency bands during N2 and N3 sleep stages to investigate the role of thalamic dysfunction in sleep slow oscillations in a patient with fatal familial insomnia. They compared this with healthy controls. MD values in the insomnia patient were significantly elevated in both thalamus and cingulate cortex, while delta (and sigma) power markedly dropped during sleep across all cortical areas. Although the relationship between elevated MD values and reduced delta power was again not directly quantified, the authors argued for an association between thalamic neurodegeneration and altered electrophysiological activity.

### Alpha Oscillations

Alpha oscillations (∼8–14 Hz) are spectral components that were already observed by Hans Berger about a century ago (Berger, 1929). For many years they were thought to ‘only’ represent idling activity of the visual cortex because they are particularly dominant at occipital EEG channels when participants have their eyes closed.

#### Resting State Studies

Valdés-Hernández et al. (2010) examined the association between the WM architecture and frequency of the alpha peak in an eyes-closed condition in healthy participants with focus on occipital electrodes. The frequency of the alpha peak was positively correlated with FA values in the posterior and superior corona radiates of both hemispheres, as well as in the isthmus and the tapetum of the corpus callosum (connecting the bilateral superior occipital lobes). By contrast, there were negative correlations with FA of the splenium and the posterior part of the corpus callosum connecting inferior occipital lobes (all correlations were significant). The sign variability in the correlation between alpha peak frequency and corpus callosum FA might be due to the dual inhibitory and excitatory role of the corpus callosum (Bloom and Hynd, 2005) or may reflect a negative effect of fiber density on conduction velocity (Reutskiy et al., 2003) since the splenium exhibits a high fiber density (Barazany et al., 2009).

Much in line with this, Jann et al. (2012) studied the relation between dMRI measures and alpha power in healthy participants in an eyes-closed condition. Mean individual alpha frequencies (m-IAF) were defined as the mean peak position within 8–12.5 Hz across recording epochs. The DTI data revealed significant positive correlations between higher IAF and increased FA values in the superior longitudinal fascicle, the genu and the splenium of the corpus callosum. These connect the core regions of the default mode network and working memory (Damoiseaux and Greicius, 2009; Jann et al., 2009). The correlations with AD (and RD) values were like those with FA and one may speculate that the increased FA values could be attributed to high AD values. Considering IAF as a marker for the global rate of information transfer between distributed networks (Klimesch, 1996), the positive relationship between FA and m-IAF reported by Jann et al. (2012) suggests faster signal transmission within thicker fibers.

As already discussed above, Duru et al. (2016) studied patients with thalamic stroke. They also analyzed alpha power at occipital electrodes and its correlation with FA values in various areas. These correlations were altered in stroke, whereby FA values in the body of corpus callosum turned out to be significantly correlated with occipital alpha power. Since the maximum correlation between alpha power and FA was located in the superior corona radiata, one may conjecture that the cortico-thalamocortical cycles are related to changes in alpha oscillations (Contreras et al., 1996; Crunelli and Hughes, 2010).

#### Task-Related Studies

Weinstein et al. (2018) studied bilateral (motor) activation in children with unilateral cerebral palsy. In many of these children, the non-affected side shows involuntary mirror movements during unimanual movement of the affected hand. In addition to an eyes-open resting state condition, EEG data were collected during a motor task (squeezing a soft plastic sponge ball). The mean alpha power was determined over the sensorimotor cortex during the resting state and during the production of unimanual movement relative to rest. Fiber tracking served to calculate DTI metrics of the corpus callosum and cortico-spinal tracts. As compared to the less affected side, the cortico-spinal tracts of the affected side showed higher diffusivity (AD and RD) and lower FA values. Children with mirror movements showed higher FA values than children without, especially in the genu, midbody, and splenium of the corpus callosum. Tractography did not reveal any significant injury abnormality in corpus callosum fibers. Yet, the EEG displayed a strong alpha-restoration after movement termination of the affected side in the hemisphere contralateral to the moving hand. Unfortunately, the study did not address direct correlations between FA values and EEG alpha power. Yet, Weinstein et al. (2018) suggested that motor activation in the less-affected hemisphere could be due to ipsilateral motor connections, simultaneous brain activation or lack of inhibition through the corpus callosum.

### Beta Oscillations

Despite the strong link of beta oscillations to motor performance, all studies collected here linked dMRI outcomes with beta power during rest.

#### Resting State Studies

As mentioned earlier, Scrascia et al. (2014) studied older adults with dementia and compared their structural and functional markers with those of age-matched healthy controls. Beta power sharply decreased in frontopolar and frontal regions in patients with mild cognitive impairment and more so in mild and moderate Alzheimer’s disease. This power drop was significantly correlated with reduced FA values in frontal WM and in the anterior region of the corpus callosum. In line with fronto-temporal disconnections related to Alzheimer’s disease, early and progressive structural damage in frontal areas seems to yield a progressive reduction of beta power (Delatour et al., 2004).

Coming back to the study by Duru et al. (2016) on thalamic stroke, beta power and FA values in the left posterior limb of the internal capsule in healthy participants displayed a significant correlation. However, in stroke patients this correlation was observed in the right external capsule and the anterior and posterior limbs of the internal capsule. Considering other studies on stroke (van Wijngaarden et al., 2016), we are tempted to speculate that the reduced beta power in stroke patients included in the work from Duru et al. (2016) correlated with decreased FA values.

### Conclusion

The combination of dMRI and EEG power at different frequencies can reveal how neural structure and function are coupled. Our review of the literature suggests that when associating with dMRI, the EEG spectral power in the lower frequency bands (delta and theta but also alpha) can be considered a functional expression of structural abnormalities, especially when applied to the resting state condition or during sleep. Analyzing dMRI together with beta power showed the sensitivity of beta power (and EEG spectral power in general) to changes in structural characteristics of cognitive and motor networks, as observed in patients with Alzheimer’s or stroke; cf. Table 2. In any case, EEG spectral power appears to be a marker for functional activity that is clearly affected by differences in structural features.

**TABLE 2:** Studies using spectral analysis, including positive (+) or negative association between spectral power in several frequency bands and DTI metrics.

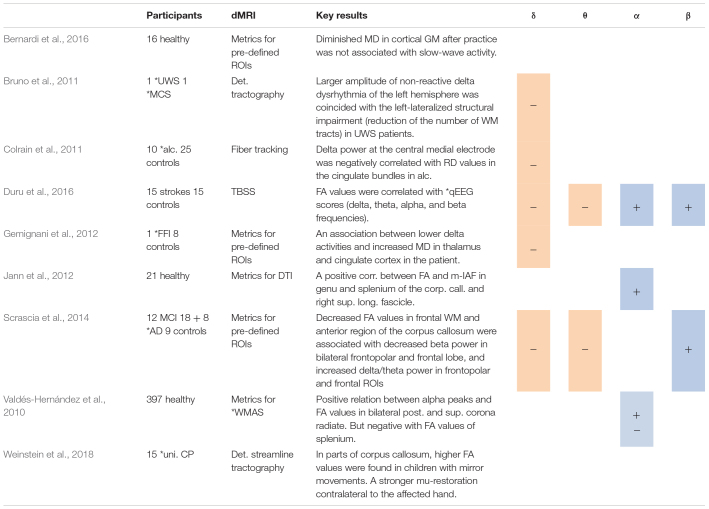

*AD, Alzheimer disease; CP, cerebral palsy; UWS, unresponsive wakefulness syndrome; MCS, minimally conscious state; FFI, fatal familial insomnia; uni. CP, unilateral cerebral palsy; alc., alcoholics; qEEG, quantitative EEG; WMAS, white matter architecture of the single bundle of fibers; MCI, mild cognitive impairment.

## Synchronization Analysis – Distant Functional Connectivity

The brain’s structural connectivity contributes to shaping neurophysiological activity, and thereby influences functional connectivity among neuronal populations (Wang et al., 2013) and brain regions (Honey et al., 2009; Deco et al., 2011). The strength of functional connectivity may depend on the microstructural organization of neuronal fibers crossing through the cerebral WM (Teipel et al., 2009; Cohen, 2011). A set of functionally connected regions is referred to as a functional network. Some functional networks can be detected during the resting state (Teipel et al., 2009), while others in the context of task-related behavior (Cohen, 2011; Liu et al., 2017) some of which persist across behavioral states like the default mode network (Cociu et al., 2018).

### Resting State Studies

Teipel et al. (2009) investigated the relationship between regional interhemispheric coherence and subcortical fiber tracts in patients with amnestic mild cognitive impairment during eyes-closed resting state. Inter-hemispheric coherence was determined for various frequency bands between bilateral pairs of electrodes. Patients and healthy controls showed associations between reduced temporo-parietal alpha coherence and reduced FA/increased MD values in a diversity of brain regions. In the patients only, reduced frontal alpha coherence between frontal pairs of electrodes was associated with reduced FA and increased MD values in anterior corpus callosum, frontal lobe WM, thalamus, pons, and cerebellum. That is, corpus callosum, thalamus, mesencephalic and pontine WM and cerebellum seem to be involved in the maintenance of inter-hemispheric alpha coherence during resting state.

Changes in phase synchronization due to mild traumatic brain injury (mTBI) and its association with structural abnormality of WM axons were estimated from eyes-closed resting state EEG (Wang et al., 2017). The (weighted) phase-lag index between several pairs of electrodes served as a measure of synchronization. Across mTBI cases, a reduced phase synchronization between the pairs of (frontal, central, and parietal) medial and (central) lateral electrodes in the lower gamma frequency band (25–40 Hz) was significantly correlated with reduced FA values in the right inferior cerebellar peduncle, showing that an impaired WM microstructural organization is accompanied by an altered synchronization in the lower gamma frequency range. In the same spirit, Sponheim et al. (2011) studied a group of healthy controls and soldiers with mTBI caused by combat-related blast. Phase locking values between pairs of scalp electrodes were estimated during eyes-closed resting state condition. The employed time-varying analysis of neural synchronization (Cohen, 1995; Aviyente et al., 2011) allowed for determining similarities between signals from distinct brain regions. FA values of posterior/anterior corpus callosum and left/right anterior thalamic radiations were averaged over voxels within each ROI. In the anterior corpus callosum, reduced FA values showed a positive correlation with reduced beta synchronization between frontal and pre-frontal electrodes. In the left anterior thalamic radiation, reduced FA values correlated positively with reduced beta/gamma synchronization between distinct frontal electrode pairs, but only in the mTBI group. Hence, it seems save to conclude that altered WM tracts characterized by reduced FA values are associated with a reduced neural synchronization in both beta and gamma frequency bands.

### Task-Related Studies

The interaction between structure and function during performance of a cognitive control task in mTBI patients was investigated by Cavanagh et al. (2019). Participants were asked to respond to two-alternative-forced choice task based on the dot pattern expectancy variant of the AX-Continuous performance task (Barch et al., 2008) as a measure of balance between proactive and reactive control. Spectral power and phase angles were estimated to quantify inter-site phase clustering between medial and lateral frontal electrodes, which subsequently was compared with FA values (Smith et al., 2004) for all regions of the Johns Hopkins University WM atlas (Mori and van Zijl, 2007). FA and left-specific inter-site phase clustering in the theta frequency band were significantly correlated in both groups. In the mTBI group, this correlation was diminished along with smaller left-specific inter-site phase clustering and fewer voxels with lower FA, suggesting frontal theta band synchrony of cognitive control to be sensitive to structural disorders.

With the aim to identify long-range structural networks that support error processing function, Cohen (2011) determined functional connectivity in healthy individuals performing an auditory-visual task that was designed to elicit response errors. Time-frequency analysis of each electrode and phase synchronization between pairs of electrodes in the theta frequency band served as a measure of functional activity/connectivity. Cohen (2011) estimated structural connectivity via probabilistic tractography with seed regions selected from dipole sources of error-related theta activity (Delorme and Makeig, 2004). Those were mostly located in the anterior cingulate cortex and surrounding tissue, much in relation with the earlier discussed ERN. The number of pathways starting from the seed region and passing through the seed voxel (coined tract strength) was considered as structural connectivity index. Finally, Cohen (2011) correlated tract strength, power, and synchronization between the medial frontal electrode and other scalp electrodes across individuals. Participants with increased theta synchronization had stronger WM connections (based on the tract strength) from dipole source locations through the corpus callosum and fibers leading to the superior frontal gyrus (dorsomedial prefrontal WM pathways). The error-related theta power at the medial frontal electrode was positively correlated with the tract strengths between seed regions and ventral striatum, motor cortex, and ventrolateral prefrontal cortex. Cohen (2011) suggested that structural networks underlie the generation of medial frontal error-related neural dynamics and the neural activity related to error processing.

Liu et al. (2017) studied theta synchronization between medial frontal and posterior parietal cortices during response conflict conditions. They collected task-related EEG data from healthy participants using a modified flanker task to eliminate feature integration and contingency learning (Duthoo et al., 2014). Participants were asked to respond to the target digit (placed in the middle of four flanker digits) by pressing the corresponding key on the keyboard. A time-frequency decomposition yielded inter-channel phase synchronization in the theta frequency band between medial frontal and posterior parietal cortex. Voxel-wise statistical analyses of DTI indices were performed. Higher conflict-induced theta synchronization between medial frontal and right parietal electrodes turned out to be negatively correlated with reduced AD values in the genu, the splenium, the body of corpus callosum, and bilateral anterior and superior corona radiates. Given the earlier suggested involvement of posterior parietal cortices during conflict conditions (Bunge et al., 2002; Brass and von Cramon, 2004), the pattern of theta synchronization was interpreted as transient functional connectivity due to the induced conflict condition.

### Conclusion

The combination of dMRI and coherence/phase synchronization allows for studying the link between functional and structural connectivity and to identify structural networks underlying task-related or resting state functional networks at different frequency bands (Table 3). All the reviewed studies support the notion that structural connectivity is associated with both task-related and resting state functional coupling at certain frequency bands. Overall, it seems that reduced coherence/phase synchronization is accompanied by an altered WM microstructural organization.

**TABLE 3:** Studies using synchronization analysis*, including positive (+) or negative (−) associations between measures for synchrony in several frequency bands and different DTI metrics.

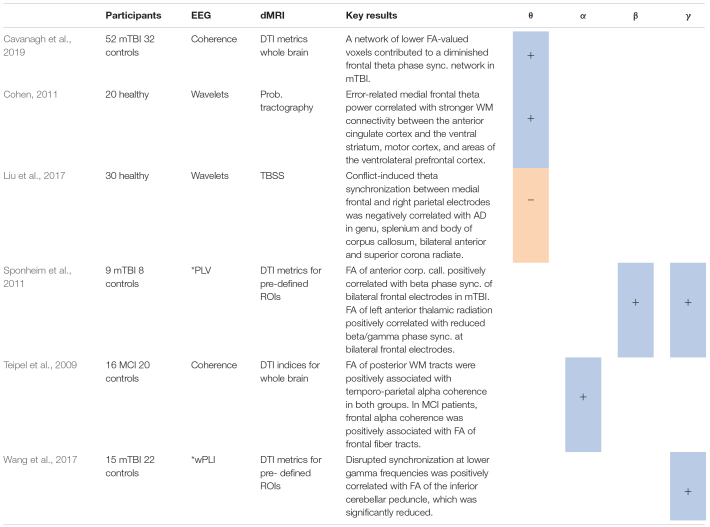

*PLV, phase locking value; wPLI, weighted phase lag index.

## Source-Level Analysis

An important aim of functional neuroimaging is to localize active brain regions. By now, there is a plenitude of methods available to estimate source activity potentially generating experimentally observed EEG. Inverse calculations yield virtual sensors or sources in the brain that may undergo analysis identical to what has been outlined in *Sections “Event-Related Potentials,” “Spectral Analysis – Local Functional Connectivity,” and “Synchronization Analysis – Distant Functional Connectivity”.*

### Resting State Studies

Arrubla et al. (2017) investigated whether diffusivity and EEG sources are correlated with magnetic resonance spectroscopy (MRS) derived glutamate levels as recorded in posterior cingulate cortex. They hypothesized that glutamate is key to the excitation/inhibition balance in the brain. Glutamate concentration in the posterior cingulate cortex was recorded using single-voxel spectroscopy in a group of healthy adults during the eyes-closed resting state condition. EEG and fMRI were collected, and cortical locations of electrical neuronal generators were estimated. From the DTI data, MD maps were constructed. Across the default mode network, glutamate concentration was negatively correlated with source activities at high alpha and low beta frequencies but was positively correlated with low alpha. Glutamate concentration also showed negative correlations with MD values within the posterior cingulate cortex. All correlations were statistically significant. Apparently, both current densities (at alpha and beta frequencies) and structural properties in the posterior cingulate cortex, a critical node in the default mode network, are mediated by variations in glutamate.

In patients with epilepsy during eyes-closed resting state, Chu et al. (2015) determined EEG source activity (Hamalainen and Sarvas, 1989) for the junction point between GM and WM related to the digitized location of each scalp electrode. Functional networks based on coherence were shown to be shaped by underlying structural connectivity, especially in the gamma frequency band (cf. Table 4 for DTI processing). Additionally, increased functional connectivity between pairs of nodes in all frequency bands was significantly correlated with increased structural connectivity between the same nodes. In other words, within every patient, the functional connectivity between nodes that were structurally connected was significantly stronger than between nodes with zero structural connectivity. This was the case for all frequency bands.

**TABLE 4:** Studies using source localization*, including positive (+) or negative (−) associations with different DTI metrics.

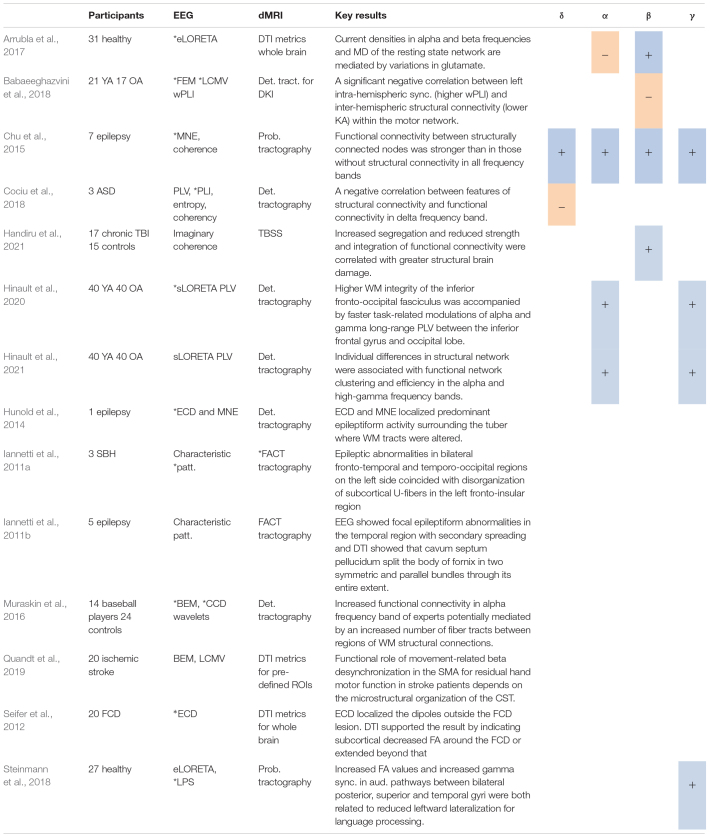

*sLORETA, standardized low-resolution electromagnetic tomography; eLORETA, exact low-resolution electromagnetic tomography; LCMV, linearly constrained minimum variance; MNE, minimum norm estimation; ECD, equivalent current dipole; CCD, cortical current density; LPS, lagged phase synchronization; SBH, subcortical band heterotopia. ASD, autism spectrum disorder; YA, younger adults, OA, older adults; BEM, Boundary Element Method, FEM, Finite Element Method; FACT, Fiber assignment by continuous tracking algorithm; Patt, pattern.

Seifer et al. (2012) targeted source locations of focal epileptogenic activities in patients with focal cortical dysplasia (FCD) during eyes-closed resting state. Equivalent current dipoles revealed neural sources of the EEG that were related to interictal spikes in regions around the focal cortical dysplasia lesion (as pre-detected by MRI). Next, DTI data were assessed using FA maps to detect WM areas with moderate or severe interhemispheric asymmetries in mean FA values. Areas with moderate or severe inter-hemispheric asymmetries were associated to the focal cortical dysplasia lesion and its surroundings. By the same token, patients with inter-hemispheric asymmetries showed decreased FA values in subcortical regions around the focal cortical dysplasia and even beyond the MRI-detected structural limits. This indicated epileptogenic zones in focal cortical dysplasia to be larger than the lesion in visible MRI. The results from EEG and DTI supported the notion that the epileptogenic zone in FCD is larger than the visible lesion in MRI, involving perilesional cortex.

Similar to the Seifer et al. (2012) study, Hunold et al. (2014) used both EEG and DTI techniques to identify the location of epileptiform activity in a single patient with tuberous sclerosis complex, which is a rare disorder of cortical tubers and is characterized by epileptic seizures. EEG signals were source localized during eyes-closed resting state, which revealed focal and extended sources of epileptiform activity in the vicinity of the calcified tuber, a disorganized area of the brain that contains abnormal cells. DTI metrics were estimated from the tracts passing through the tuber, the normal appearing WM (contralateral to the tuber), the set of adjacent dipoles within the epileptogenic zone, and the contralateral dipole cloud. This revealed changes in WM microstructural organization (lower FA and higher RD values) for the tracts passing through the region localized by equivalent current dipole, in the vicinity of the tuber. That is, epileptiform activity in tuberous sclerosis complex patients may ‘originate’ from an abnormally developed cortex around the tubers.

Iannetti et al. (2011b) evaluated the role of the cavum septum pellucidum and fornix in the generation of epilepsy and seizures. EEG during the eyes-closed resting state revealed focal epileptiform abnormalities in temporal regions with secondary spreading. DTI tractography showed that the cavum split the body of the fornix in two symmetric and parallel bundles through its entire extent. One may interpret this in that the septum inhibits the occurrence of epileptic discharges, and that the degeneration of this neural structure contributes to an increase in epileptic activity. The same research group also sought to characterize a rare condition called neuronal migration disorder, which is also known as subcortical band heterotopia and often characterized by epilepsy (Iannetti et al., 2011a). The disorder is a malformation of cortical development in which GM is bilaterally misplaced in WM parallel to the surface of the neocortex. In three patients, DTI data were analyzed via tractography (Mori et al., 2002) to reconstruct occipital and fronto-insular U-fibers. Eyes-closed resting state EEG recording showed spike and epileptic abnormalities in bilateral fronto-temporal and temporo-occipital regions, particularly on the left side. Tractography revealed a reduction and disorganization of subcortical U-fibers, particularly in the left fronto-insular region, and, in one patient, in the occipital region. Unfortunately, no subsequent correlation analysis was conducted. Yet, the results suggest that in the left hemisphere, the seizures arising from the heterotopic GM were accompanied by altered WM connectivity, leading to a functional isolation of the normotopic cortex with apoptotic processes.

Cociu et al. (2018) investigated aberrant neural information exchange in children with autism spectrum disorder. They estimated EEG sources both via ‘simple’ dipole fitting (Delorme et al., 2012) during eyes-open resting state. Functional connectivity was estimated between dipoles close to fMRI pre-localized regions including the precuneus, posterior cingulate cortex, left/right parietal cortex, superior frontal gyrus and medial prefrontal cortex, which all form part of the default mode network. Functional coupling based on various measures was estimated in various frequency bands between all pairs of sources. Volume, tract length and number of tracts were determined between the same pairs (see Table 4 for details). Cociu et al. (2018) could identify significant negative correlations between the phase locking value in the delta frequency band and tract length/volume between regions of the default mode network.

### Task-Related Studies

Babaeeghazvini et al. (2018) studied correlations between structural and functional connectivity and their association with bimanual coordination as a function of age. Sources were reconstructed with a focus on the beta frequency band (Van Veen et al., 1997) and unraveled significant contributions of bilateral primary motor cortex and dorsal premotor areas. From the resulting source signals, inter- and intra-hemispheric functional connectivity was estimated using the weighted phase-lag index. Kurtosis anisotropy values and the standard deviation of kurtosis (Poot et al., 2010) at the fiber tracts between the same atlas-based regions were determined (cf. Table 4). In older adults, phase-lag values between left/right dorsal premotor and left primary motor cortex were significantly correlated with KA values in bilateral primary motor cortices. Overall, older adults showed both increased functional connectivity and reduced structural connectivity for all connections when compared to younger adults. These results suggested a diminished structural connectivity to be associated with an increased functional one in older adults.

More recently, Hinault et al. (2020, 2021) studied the effects of age-related changes in the WM microstructural organization on dynamic functional coupling between brain regions during performance of an arithmetic verification task. They focused on the moment after a working memory cue for an arithmetic operation, the subsequent delay period, and the time around an arithmetic problem displayed on a screen. Hinault et al. (2020) estimated phase-locking values (Lachaux et al., 1999) between bilateral frontal-occipital fasciculi, superior longitudinal fasciculi, and the cingulum bundles. Later on, Hinault et al. (2021) extended the analysis to source reconstructed signals in the regions of the Desikan atlas (Desikan et al., 2006) to quantify whole-brain functional network organization using phase-locking values. Both studies performed DTI tractography between the same regions as used for functional coupling, however, the approach to assess structural-functional relationships differed. Hinault et al. (2020) performed a mediation analysis for the effect of task-relevant functional-structural couplings on behavioral performance, whereas Hinault et al. (2021) used graph theory to estimate individual structural clustering levels and their relationship to task-related functional networks. In the first study, they found a mediation effect of dynamic functional connectivity and WM microstructure in the tract connecting inferior frontal cortex and the occipital lobe over behavioral performance. In older adults, a relatively preserved WM microstructural organization was accompanied by earlier and greater frontoposterior functional coupling which together mediated lower interference effects on the arithmetic verification task. On the other hand, Hinault et al. (2021) found that lower structural clustering in older adults correlated with reduction in clustering and efficiency of task-related functional network in alpha and high-gamma frequency bands. Taking together, the results suggest that age-related changes in WM microstructure affect long-range dynamic functional coupling and stability of functional network.

Likewise recently, Handiru et al., 2021 investigated the graph-theoretical properties in functional connectivity estimated between EEG source signals in two groups of chronic TBI patients and controls during a balance perturbation task. They used the imaginary part of coherence in theta, alpha and beta frequency bands to measure the functional connectivity between ROIs. Graph-theoretical measures served to quantify global network properties. Higher FA and lower MD values were significantly associated with higher strength of functional connectivity in beta frequency band during a postural control task. The result is in line with study by Chu et al. (2015), who reported the association between structural connectivity and EEG source-based functional connectivity in higher frequency bands (beta and gamma); see above.

Muraskin et al. (2016) compared two groups of collegiate baseball players (experts) and novices (controls) searching for differences in both functional and structural connectivity. Participants performed a surrogate baseball pitch Go/No-Go task prior to an eyes-open resting state scan during which simultaneous fMRI-EEG and DTI data were collected. Using a cortical current density source model (He et al., 2011), alpha and beta power were estimated at source level in left and right supplementary motor areas (Muraskin et al., 2015). In addition, functional connectivity was estimated between alpha power from right supplementary motor area, as the seed region, and BOLD time series from source locations in non-supplementary motor area voxels (Muraskin et al., 2017), followed by deterministic tractography. Significant correlations were found between alpha power at the right supplementary motor area and the BOLD signal at left superior frontal gyrus and left insula. Correlations were negative in experts and positive in controls. There were also positive correlations in experts and negative ones in controls between alpha power at right supplementary motor area and BOLD at right middle temporal gyrus. And there was a negative correlation in experts (positive in controls) between beta power at right supplementary motor area and BOLD at left superior frontal gyrus. Moreover, experts displayed an increase in functional connectivity of alpha frequency between supplementary motor area and cerebellum. Compared to controls, experts exhibited a significantly larger number of structural connections (fiber tracts) between right superior frontal gyrus and left posterior cingulate, right inferior frontal gyrus and right cerebellum, and right precentral gyrus, and both left posterior cingulate and left insula. On the other hand, controls exhibited a significantly larger number of structural connections between right supplementary motor area and right inferior frontal gyrus and left superior frontal gyrus and left insula. Here, we would like to note that Muraskin et al. (2016) did not correct significant levels for multiple comparisons. We also note that Muraskin et al. (2016) failed to establish a direct structural connectivity between supplementary motor areas and the selected regions. Nonetheless, the observed functional relationships might be supported by indirect structural connections. In fact, Muraskin et al. (2016) suggested the functional connectivity relationship between alpha power in the supplementary motor area and the BOLD signal in the right cerebellum to be mediated by structural connections from right supplementary motor area to right inferior frontal gyrus, and then right inferior frontal gyrus to right cerebellum.

Steinmann et al. (2018) collected task-related EEG from healthy participants during a dichotic listening task to study the association between language lateralization and structural connectivity of interhemispheric auditory pathways, as well as gamma-band synchrony between bilateral auditory cortices. Two different consonant-vocal syllables were presented to each ear simultaneously and participants reported the best perceived syllable after each trial. EEG source reconstruction was performed (Pascual-Marqui, 2007) and functional connectivity was estimated as (lagged) phase synchronization in the gamma frequency band (30–100 Hz) between Heschl’s gyrus and posterior superior temporal gyrus (Pascual-Marqui et al., 1994). For the DTI, ROIs were chosen according to the ones used for functional connectivity, including Heschl’s gyrus, posterior superior temporal gyrus and the mid-sagittal mask of the corpus callosum. Probabilistic tractography served to extract inter-hemispheric auditory pathways by choosing right Heschl’s gyrus as seed mask and left Heschl’s gyrus as termination mask passing through the corpus callosum. The same procedure was applied to extract the tracts between left and right posterior superior temporal gyri. Mean FA values were determined for the obtained tracts. With this, Steinmann et al. (2018) found that increased FA values and increased gamma synchronization in inter-hemispheric auditory pathways between bilateral posterior superior temporal gyri were both related to a reduced leftward lateralization for language processing. However, the relationship between FA values of inter-hemispheric auditory pathways and lagged phase synchronization values in the gamma frequency band did not reach significance.

To investigate hand motor outcome in chronic stroke patients, Quandt et al. (2019) combined information of oscillatory changes during specific grasping movements and the microstructural organization of the corticospinal tract. Participants executed repetitive reaching and grasping movements with their affected hand under two conditions: (i) a pinch grip, where participants lifted a weight with their affected thumb and index finger; and (ii) a hand grip, where the weight was lifted with all fingers. EEG sources for primary motor cortex, ventral premotor cortex, and supplementary motor area were reconstructed (Van Veen et al., 1997). ROIs were pre-selected based on a previous fMRI study on hand grip in healthy older adults (Schulz et al., 2016). Spectral power was estimated in the theta, alpha and beta frequency bands during the pinch grip condition, whole mean FA values were determined for the left and right corticospinal tract from the mesencephalon to the cerebral peduncle. Linear regression revealed that beta power in the ipsilesional supplementary motor area and FA values of the corticospinal tract, on the same side, best explained residual motor function of the affected hand. The association between movement-related beta desynchronization in supplementary motor area and motor impairment suggests direct descending motor control from pyramidal neurons. The findings suggest that the microstructural organization of corticospinal tract determines the degree of the functional involvement of the supplementary motor area during motor performance in stroke.

### Conclusion

The EEG source localization yields (virtual) brain activity at voxel level. When combined with dMRI one may estimate structural connectivity between the functionally active brain regions. This may reveal the underlying structural connectivity of the functional networks. For example, Cociu et al. (2018) found that structural connectivity agrees with resting state functional connectivity in the delta frequency band within the default mode network. EEG source localization also allows for estimating the regions with abnormal functional activity (e.g., epileptiform activity), which often agree with structural abnormalities as reflected in different dMRI outcome measures (Seifer et al., 2012; Hunold et al., 2014).

## Discussion

We reviewed a variety of studies that combined EEG with dMRI. Before discussing them, we would like to note that despite the considerable insights into the relationship between GM/WM microstructural organization and neural functioning, the combination of EEG and dMRI comes with many challenges, often involving defining details of the measurement procedure and the analysis. While there are several technical hurdles to take, one must realize that the major bottleneck in this field is that most studies are low-powered, sometimes covering only a handful of patients with a very specific phenotype. We highlighted this within the current review but realized that the generalizability of many findings remains limited and calls for more research. Yet, in the following we briefly recapitulate some of the insights gained from the here-reviewed studies and put them in a broader context of relating functional and structural properties of the brain.

### Response Timing and Amplitude Relate to White Matter Microstructural Organization

With respect to studies using event-related designs, most of the findings can be explained by accounting for the conceptual differences in EEG outcome measures – latency vis-à-vis amplitude changes – and their relation to neural processing. In line with a mechanistic view (see Section “A Mechanistic View”), changes in the WM microstructural organization affect the rate of information transfer (e.g., visual and auditory information) between different brain regions. ERP latencies do address this information transfer directly. Hence, one may expect a close relationship between conduction delays and the structural properties of WM measured via dMRI (Cuypers et al., 1995; Kesselring, 1997). Apparently, a variation in WM microstructural organization is closely related to hemispheric timing differences during auditory or visual task performances. The Westerhausen et al. (2006) and Whitford et al. (2011a) studies confirmed this notion by showing a negative correlation between inter-hemispheric transmission time (difference between left and right P100 latencies at occipital electrodes) and MD values in the posterior corpus callosum and FA values in the callosal fibers. Occipital P100 peak latencies are related to primary visual information processing situated in the extrastriate cortex (Gomez Gonzalez et al., 1994; Di Russo et al., 2005). Interhemispheric connections between regions of the striate cortex are few and far between. As such, one may consider the P100 as the first visual processing step within the ipsilateral hemisphere.

Evstigneev et al. (2013) reported a negative correlation between decreased FA and increased latency of the P300 peak in the anterior quadrant of the epileptogenic hemisphere. Earlier ERP studies revealed that the P300 generation involves interhemispheric integration of sensory information (Knight et al., 1989; Yamaguchi and Knight, 1991, 1992). That is, P300 appears to be related to structural connectivity between the two cerebral hemispheres (Caminiti and Sbriccoli, 1985; Pandya and Seltzer, 1986). If true, the very fact that the corpus callosum is critical for interhemispheric transmission efficacy (Iacoboni and Zaidel, 1995; Nowicka et al., 1996) may explain why the P300 latency depends on the microstructural organization of the corpus callosum. The negative correlations between reduced hemispheric N1 latency differences and increased FA values in the posterior callosal third reported by Friedrich et al. (2017) support this interpretation: microstructural properties of the corpus callosum modulate the timing differences between hemispheres.

Changes in WM microstructural organization also affect amplitude of ERP components. Higher ERP amplitudes appear to be related to WM microstructure, as, e.g., shown by the positive correlation between N2 amplitudes and FA in the superior longitudinal fascicle linking the parietal and frontal cortices (Gao et al., 2017), or by a positive correlation between the (strengths of the) ERN amplitude and higher FA values in the posterior part of the left cingulum bundle reported by Westlye et al. (2009).

### Electro-Encephalography Power Might Be Associated With Structural Degeneration

In contrast to the studies on event-related potentials, the findings on spectral EEG content are more diverse, as we found both positive and negative correlations, albeit not always significant. Slow wave power changes were often investigated in the context of neuropathology to identify associations between abnormal functional activity (in delta and theta but also low-alpha frequency bands) and structural abnormalities underlying a specific disorder. All studies claimed a relationship between neurodegeneration and spectral power, indicating that in combination with dMRI the spectral power may serve to identify structural disorder. For instance, the observed reduction of power across cortical areas which coincided with elevated MD in the thalamus and the cingulate cortex in a fatal familial insomnia patient (Gemignani et al., 2012) supports the importance of the thalamus in the generation of low-frequency events, at least during sleep (Contreras et al., 1996; Crunelli and Hughes, 2010). Cortico-thalamocortical cycles are known to relate to changes in alpha frequency (Contreras et al., 1996; Crunelli and Hughes, 2010). Interestingly, such associations also extend to even higher frequency bands. An example is given by Inoue et al. (2012), where DWI revealed anatomical connections between the lesioned thalamic site and premotor cortex, primary motor cortex and primary sensory area. Beta power increased in frontal, central and centroparietal electrodes related to the hand area of the sensorimotor cortex. The lesioned thalamic nuclei might thus be responsible for the abnormally increased beta activity in the sensorimotor cortex, as well as excessive cortical inhibition.

Valdés-Hernández et al. (2010) reported positive correlations between the alpha peak frequency and FA values in the posterior and superior corona radiate, and in the isthmus and tapetum of the corpus callosum. However, they also reported negative correlations in the splenium and the inferior part of the corpus callosum. The different sign in the correlation between alpha peak frequency and FA in the corpus callosum might be due to the dual inhibitory and excitatory role of the latter (Bloom and Hynd, 2005). Yet, it might also reflect a negative effect of fiber density on conduction velocity (Reutskiy et al., 2003) given that the splenium exhibits a high fiber density (Barazany et al., 2009). Either way, the elevated degree of FA values probably indicates a highly organized directionality of fiber bundles and axon density in the corona radiate, splenium of the corpus callosum, and major forceps (Mädler et al., 2008; Friedrich et al., 2020). Hence, the relationship between the alpha peak frequency and FA value underscores the importance of WM architecture for the emergence of alpha oscillations in the ROIs, at least in some cases.

Scrascia et al. (2014) reported strong correlations between decreased beta power in frontopolar and frontal regions and decreased FA values in frontal WM and anterior regions of the corpus callosum in patients with mild cognitive impairment and Alzheimer’s disease. The latter are indicative for early fronto-temporal structural disconnections due to the progression of Alzheimer’s disease (Delatour et al., 2004). Previous studies (Jeong, 2004; Babiloni et al., 2006) already reported a shift of the power spectrum to lower frequencies and a decrease in coherence of higher frequencies as the hallmark of electrophysiological abnormalities and a reflection of early and progressive structural damage.

### Linking Electro-Encephalography Sources and Connectivity to Structure – New Trends

The more recent studies on functional connectivity largely confirm the findings on local power changes. They demonstrate a close relationship between structural and functional connectivity both in resting state and during task execution. In mild cognitive impairment patients, frontal alpha coherence and FA values were positively correlated in frontal fiber tracts (Teipel et al., 2009), while in patients with mild traumatic brain injury a likewise positive correlation was found between beta phase synchronization in frontal regions and FA values in the anterior corpus callosum and in the left anterior thalamic radiation (Sponheim et al., 2011). The latter suggests a weak association with frontal function due to damaged WM tracts that is also manifested in a positive correlation between reduced phase synchronization at low gamma frequencies and reduced FA values in the right inferior cerebellar peduncle (Wang et al., 2017). This supports the role of gamma frequency in the integration of sensory processing and sensory-motor coordination (Womelsdorf and Fries, 2006; Senkowski et al., 2008). Increased conflict-induced theta synchronization between medial frontal and posterior parietal cortices was negatively correlated with reduced AD values in the genu, the splenium, the body of the corpus callosum, and the bilateral anterior and superior corona radiate (Liu et al., 2017). This may reflect a compensatory increase in functional connectivity due to a reduced microstructural organization (de Kwaasteniet et al., 2013). Recall here that an increased axonal diameter increases the conduction velocity of action potentials and this correlates positively with AD, referring to the magnitude of diffusion parallel to fiber tracts (Takagi et al., 2009).

While in many studies the EEG source localization merely served to select seed regions for DTI tractography (Chu et al., 2015; Muraskin et al., 2016; Cociu et al., 2018), it has also been applied to identify a direct relationship between current densities of different frequency bands and structural properties (Arrubla et al., 2017). The combination of EEG source localization with dMRI has been particularly fruitful in studies on pathology. They revealed the location with abnormal functional activity to be very close to the location where abnormal structural properties are reported. By this, Seifer et al. (2012) could identify epileptogenic zones to be larger than the visible lesion identified by MRI and Hunold et al. (2014) could conjecture that epileptiform activity in patients with tuberous sclerosis complex has its origin around the tubers.

### Comparison Between Electro-Encephalography and Functional Magnetic Resonance Imaging

Compared to fMRI, EEG has several advantages which may help for better understanding the relationship between structural and functional properties. First, EEG records electrophysiological activity at a high temporal resolution that can be crucial for monitoring rapid, functional changes. Second, EEG does not necessarily have to be recorded in a lab-setting. In fact, some current EEG systems are portable, which allows for recording functional brain activity during daily tasks (Bruijn et al., 2015; Park et al., 2015). Yet, it appears that many EEG and fMRI studies do converge when it comes to source localization and estimating functional connectivity. For example, EEG (Taylor et al., 2007; Cohen, 2011) and fMRI (Van Veen and Carter, 2002) studies agree when studying the involvement of anterior cingulate cortex in error processing and generation of event-related negativity. A similar agreement between EEG (Miltner et al., 2003; Debener et al., 2005; Wang et al., 2005; Cohen et al., 2008; Vocat et al., 2008) and fMRI (Van Veen and Carter, 2002; Mathalon et al., 2003) is visible when investigating pre-supplementary motor area or anterior cingulate cortex as the source of medial frontal theta activity. And, this extends to studies on the default mode network (Cannon et al., 2011) and resting state network (Samogin et al., 2020), where EEG coherence turns out to be consistent with (the spectral power of) BOLD (Ko et al., 2011). Both modalities have shown similar, age-related increases in functional connectivity (Solesio-Jofre et al., 2014; Babaeeghazvini et al., 2018). With respect to the link between functional and structural properties, both EEG (Westlye et al., 2009; Gao et al., 2017) and fMRI (Olesen et al., 2003; Seghier et al., 2004; Persson et al., 2006; Schlősser et al., 2007) studies show correlations between dMRI measures and functional activity during similar types of cognitive performance.

### More Recent Developments

Increasing the resolution of data acquisition may lead to deeper insights into how functional and structural properties of the brain are coupled. Dense EEG arrays (i.e., 128 or even 256 electrodes) can improve source localization by increasing the signal-to-noise ratio to detect functional networks (Liu et al., 2018), potentially offering more reliable results about functional connectivity, if corrected for volume conduction effects. As for dMRI, high-angular multi-shell diffusion acquisitions may improve sensitivity and directional specificity. Such leveraging of multiple shells (i.e., *b*-values) with large numbers of diffusion directions can elicit differential tissue responses that are useful to model in greater detail WM microstructural organization. Interestingly, the impact of in-scanner motion on multi-shell measures of microstructure appears to be reduced, at least on a global level (Pines et al., 2019).

In addition, more advanced methods of analysis can greatly improve the accuracy of outcome measures. For example, even though the DTI model has clinical relevance, it is not suitable for complex fiber configurations, such as crossing and ‘kissing’ fibers (Jeurissen et al., 2013). Multi-compartment models like the composite hindered and restricted model of diffusion (Assaf and Basser, 2005) and the neurite orientation dispersion and density imaging model (Zhang et al., 2012), provide greater specificity when relating the diffusion signals to underlying WM microstructure and to functional connectivity (Horowitz et al., 2015; Deligianni et al., 2016). Furthermore, Jelescu et al. (2020) discussed different biophysical models for diffusion in brain tissue and their implications for clinical use. In recent years, WM tractography has also been improved, e.g., in better resolving multiple fiber crossings. Constrained spherical deconvolution (Tournier et al., 2004) better estimates fiber orientation distribution, and has led to the development of fiber bundle specific measures (Raffelt et al., 2017), such as apparent fiber density. The latter, so-called fixel-based analysis (FBA), can provide information about fiber density, fiber cross section and a combination of both measures.

In very recent years, an increasing number of studies extended the combination of encephalographic assessments and diffusion-based imaging with electrically or magnetically stimulating. This includes transcranial magnetic stimulation, direct current stimulation as well as deep brain stimulation. This field is rapidly growing and certainly worthwhile monitoring. For the sake of legibility, we provide a glance on this literature as a Supplementary Material – a glance because we cannot guarantee completeness.

A review entitled “Brain structural and functional connectivity” cannot ignore the developments on network assessments using graph theoretical approaches. The field is vast, and we consider a proper overview to be beyond the scope of our review. We summarized two recent studies employing graph theory (Handiru et al., 2021; Hinault et al., 2021), which has become particularly popular for quantifying connectivity (He and Evans, 2010; Sporns, 2011; Alexander-Bloch et al., 2013). Graph theory progressed toward the assessment of pathological changes, especially in functional networks (Bassett and Bullmore, 2009; Fornito et al., 2015). More recently, first steps have been taken to integrate two (or more) modalities (Guye et al., 2010; Glomb et al., 2020; Wirsich et al., 2021) also using multilayer networks (Boccaletti et al., 2014; Muldoon and Bassett, 2016; Lacasa et al., 2021). In fact, several studies that matched our original literature search criteria already followed this route but have been excluded here because they solely address the methodological advance. As such it seems just a matter of time that studies combining EEG with dMRI in ‘real’ experiments will capitalize more on the strengths of these powerful techniques.

Last but not least we would like to anticipate our plea for standardizing approaches and methods (see our general conclusions below). One step toward standardization is to provide open access to both data and analysis. Given the very nature of the topic under research, privacy-related issues may limit availability of data. Yet, there are quite recent initiatives to provide openly accessible data sets, like the Healthy Brain Network (HBN) (Alexander et al., 2017), OpenNeuro^1^, Brain Imaging Data Structure (BIDS)^2^ or John Richard’s neurodevelopmental MRI database^3^. This list could readily be extended, but again we consider digging deeper into these exciting developments beyond the scope of our current review. When it comes to analysis tools, the list is even longer, ranging from EEGLAB (Delorme and Makeig, 2004), Fieldtrip (Oostenveld et al., 2011), Statistical Parametric Mapping (Litvak et al., 2011), Brainstorm (Tadel et al., 2011), MNE-python (Gramfort et al., 2013), and NUTMEG (Hinkley et al., 2020) to ExploreDTI (Leemans et al., 2009), FMRIB Software Library (FSL) (Smith et al., 2004; Woolrich et al., 2009), and MRtrix (Tournier et al., 2019). We clearly advocate their use as they seem to converge in validity and reliability; see, e.g., (Jaiswal et al., 2020).

## General Conclusion

We reviewed various studies that combined EEG and dMRI assessments to relate functional activity with structural connectivity. The variety of techniques, methods and outcomes renders comparison a challenge, especially when it comes to the additive value of combining EEG with dMRI. Yet, the different ERP analyses revealed clear associations with dMRI measures. Apparently, the speed and strength of broadcasting between distant brain regions quantified by, e.g., ERP latencies and amplitudes can largely express the WM microstructural organization. This can be expected when adopting a simple mechanistic standpoint, i.e., that an increased WM microstructural organization and/or structural connectivity strength ‘simply’ facilitates neuronal information transfer. The EEG’s spectral power and synchronization appeared less sensitive unless used to assess the effects of neurodegenerative diseases. Nonetheless, when combined with dMRI, properties of the WM microstructural organization may eventually yield proper biomarkers for functional decline accompanying structural changes. Yet, the diversity of findings requires a very careful interpretation of the results and highlights the need to standardize EEG analysis. This is certainly true when searching for a more general relationship between functional and structural characteristics of the human brain, let alone when aiming for employing EEG-dMRI co-registration in the clinic.

## Author Contributions

PB and AD designed the study and reviewed all the included manuscript. LR-D reviewed in particular the EEG studies. JG and SS focussed on the dMRI studies. All authors wrote the manuscript.

## Conflict of Interest

The authors declare that the research was conducted in the absence of any commercial or financial relationships that could be construed as a potential conflict of interest.

## Publisher’s Note

All claims expressed in this article are solely those of the authors and do not necessarily represent those of their affiliated organizations, or those of the publisher, the editors and the reviewers. Any product that may be evaluated in this article, or claim that may be made by its manufacturer, is not guaranteed or endorsed by the publisher.

## Footnotes

1
https://openneuro.org

2
https://bids.neuroimaging.io/

3
https://jerlab.sc.edu/projects/neurodevelopmental-mri-database/
