# Supplementary material for: Brain Structural and Functional Connectivity: A Review of Combined Works of Diffusion Magnetic Resonance Imaging and Electro-Encephalography
Source: Front Hum Neurosci. 2021 Oct 7;15:721206. doi: 10.3389/fnhum.2021.721206 (PMC8529047; doi:10.3389/fnhum.2021.721206)
Supplement: Supplementary file 1 [file Data_Sheet_1.pdf]

S1 Neuro-stimulation

Another approach to probe the structure/function relationship is by electrically or magnetically stimulating the central nervous system. We briefly sketch some approaches using *transcranial magnetic stimulation* (TMS), *transcranial direct current stimulation* (tDCS), and, in the case of epilepsy, *deep brain stimulation* (DBS). In brief, if applied above primary motor cortex, single pulse TMS can elicit motor-evoked responses that can be identified via conventional electromyography. TMS has been applied repeatedly to both facilitate or inhibit the excitability of the cortico-spinal tract, dependent on the frequency of TMS repetition. tDCS is a presumably non-invasive neurostimulation technique which transmits a weak current through the brain scalp to alter the cortical excitability on a specific brain region. DBS is an invasive targeted surgical procedure in which a neurostimulator is placed inside the brain to send electrical impulses to specific neural targets.

S1.1 Transcranial magnetic stimulation

Groppa et al. (2013) studied the cortico-cortical coherence and the structural substrate of this functional connectivity in healthy participants. They estimated inter-and intra-hemispheric coherence in alpha and beta frequency bands during an eyes-closed resting state condition. Coherence was determined between electrodes over motor areas. To delineate the WM structure, seed regions were selected from significant voxels. FA values of tracts between bilateral primary motor cortices and between the stimulated motor cortex and its contralateral thalamus displayed significant positive correlations with a TMS-induced increase in alpha coherence between central electrodes. It seems that transcallosal pathways but also contralateral thalamus and thalamocortical fibers mediate stimulation-induced changes in functional connectivity between central regions.

In a similar vein, Amico et al. (2015) investigated the interplay between TMS-induced functional connectivity at the source level and structural connectivity during eyes-closed resting state in a cohort of healthy participants. Single-trial distributions of electrical sources were estimated, and reconstructed time series for all resulting dipoles were averaged over each region of the automated anatomical labelling atlas. Subsequently, the spectrum-weighted adaptive transfer function served to quantify direct functional connectivity between every pair of homologous ROIs. For the DTI data, probabilistic tractography was conducted between the same regions. After a TMS pulse, the correlation between structural and directed functional connectivity significantly increased over time in high beta and gamma frequency bands in premotor cortex contralateral to the stimulation site. However, at the whole-brain network level, the relationship between structural and direct functional connectivity decreased after a TMS pulse within each of the frequency bands. The results suggest that TMS-induced interaction between structure and function of the brain depends on the frequency at which functional connectivity is estimated and the anatomical feature of cortical region stimulated by TMS.

Bashir et al. (2012) adopted a whole-brain voxel-wise comparison of WM based on dMRI metrics to examine structural disorders related to observed abnormal EEG activity. They investigated long-lasting structural and functional impairments in a single patient with mild traumatic brain injury two and six weeks after injury, and his test scores were compared with a group of healthy controls. Topographical EEG maps were estimated for eyes-closed resting state EEG data and a voxel-wise analysis of FA values was applied to the DTI data. With respect to controls, the patient showed less intense and more widespread activation 50 ms after single-pulse TMS over left primary motor cortex in the second week. After continuous theta burst stimulation, this activation even increased, and the patient showed a significant facilitation of motor-evoked potentials. However, in week six the patient displayed a normal inhibition of motor-evoked potentials and no strong increase in brain activity. TBSS revealed no significant differences in FA values between the patient and controls. Apparently, there was a transient alteration of brain functional activity after brain injury independent of the WM microstructural organization.

Inoue (2012) investigated the (generation of) asterixis, a type of negative myoclonus. They estimated the spectral power as a function of time for a wide range of frequencies from 0.016 to 100 Hz and focused on the silent period (average of EEG data time-locked to the asterixis) during eyes-open resting state. Tracts were defined between thalamus and seven cortical regions, (prefrontal cortex, pre- and primary motor cortex, primary somatosensory cortex, posterior parietal cortex, occipital cortex and temporal cortex). The connection probability between each seed voxel in the thalamus and each cortical mask was considered as an index of structural connectivity. The change in power over time showed an increased beta power time-locked to asterixis in frontal, central and centroparietal electrodes contralateral to the affected hand and related to the hand area of the

sensorimotor cortex starting about 0.05 s prior to the onset of the silent period. If beta power in resting state reflects an excessive inhibition related to sustained hand posture, the thalamic asterixis may have been mediated by the sensorimotor hand area. Interestingly, the tractography revealed connection from lesioned thalamic parts to premotor cortex, primary motor cortex and primary somatosensory cortex. Inoue (2012) speculated that the lesioned thalamic nuclei, especially the ventral lateral nucleus, might be the cause of abnormally increased beta activity in the sensorimotor cortex and that excessive cortical inhibition may cause asterixis.

S1.2 Transcranial direct current stimulation.

Nakamura-Palacios et al. (2016) investigated structural and functional changes in alcohol or crack-cocaine addicts after repetitive bilateral tDCS over dorsolateral prefrontal cortex in two groups of real-tDCS and sham-tDCS. Participants were asked to perform a visual cue-reaction paradigm during EEG recording. Low-resolution brain electromagnetic tomography served to identify the highest changes after tDCS treatment as compared to the baseline during the P300 interval (300-500 ms). DTI data was collected before and after tDCS treatment for both groups of real and sham stimulation. The tDCS treatment was performed up to five sessions daily or every other day, each treatment lasted 20 min (2 mA) or in double applications it lasted 13 min with 20 min interval in between. FA values were determined for fibers between the ventral medial pre-frontal cortex and the nucleus accumbens in both hemispheres. The nucleus accumbens and the ventral tegmental area, along with medial prefrontal cortex, are brain structures that constitute the brain reward circuitry (Nestler 2004; Nakamura-Palacios 2011). The functional interaction between dorsolateral prefrontal cortex and ventral medial pre-frontal cortex provides an adequate self-control for decision making between competing outcomes preferences (Rudorf and Hare 2014). Ghazizadeh et al. (2012) suggested that ventral medial pre-frontal cortex controls the inhibition of action via the nucleus accumbens shell. In the real-stimulation group, ventral medial pre-frontal cortex appeared to be the brain region with the highest drug-related activity change within the P300 segment after treatment in both alcohol and crack-cocaine users. In the sham-stimulation group the highest activated region was middle temporal gyrus for alcohol addicts, and the inferior parietal lobule for crack-cocaine users. The result for the real-stimulation group was further supported by DTI, which showed increased FA values of fiber tracts between pre-frontal cortex and nucleus accumbens in the left hemisphere after treatment in crack-cocaine users with real stimulation.

S1.3 Deep brain stimulation

To pinpoint anti-seizure effects of centro-median thalamic nucleus in patients with intractable epilepsy, Kim et al. (2017) stimulated nucleus at a low frequency (5 Hz) and focused on the propagation pattern of cortical recruitment responses. Low-frequency stimulation of the thalamic nuclei creates cortical recruitment responses (Zumsteg, Lozano, and Wennberg 2006; Zumsteg et al. 2006). EEG data were collected during eyes-open resting state during 2-5 min stimulation, followed by a 2 min resting period. Subsequent EEG source localization identified the areas with maximal cortical recruitment responses in the left cingulate gyrus and the right medial frontal cortex during stimulation of the right and left centro-median thalamic nuclei, respectively. Probabilistic tractography of the DTI data revealed the existence of fiber tracts between these nuclei and EEG source localized regions (medial frontal gyrus and anterior cingulate cortex). The tract of anterior cingulate cortex propagated bilaterally to the frontal cortex, suggesting that the anterior cingulate cortex could be a hub structure for initiation and propagation of ictal discharges that could be blocked with centro-median thalamic nuclei stimulation. Deep brain stimulation of centromedian thalamic nucleus might modify the neural network and affect activity through thalamo-prefrontal and -cingulate connections.

S1.4 Conclusions

The combination of TMS and EEG modalities quantifies cortical connectivity patterns via power spectral estimates, coherence, and phase synchronization. The combination of EEG-TMS with dMRI helps identifying structural networks that are associated with (functional) connectivity, here induced by TMS. This field is rapidly developing. In the last twelve months or so, seven new studies appeared that we here list for the sake of completeness: (Bortoletto et al. 2021; Levy-Lamdan et al. 2020; Momi, Ozdemir, Tadayon, Boucher, Shafi, et al. 2021; Momi, Ozdemir, Tadayon, Boucher, Di Domenico, et al. 2021; Pinter et al. 2021; Weichart et al. 2020; Whitmer et al. 2012). As such one can expect that the combination of EMG, dMRI and neuro-stimulation will soon provide further insight into the interplay between structural and functional connectivity.

Table S1: Selected studies with neurostimulation.

	participants	EEG	dMRI	Key results
Amico et al. (2017)	14 healthy	swADTF	Probabilistic tractography	A temporary decrease in correlation between directed functional connectivity and structural connectivity after TMS stimulation. Structure/function correlation significantly increases over time in the premotor area contralateral to TMS stimulation.
Bashir et al. (2012)	1 TBI<br>12 controls	Topographical EEG maps	TBSS	In the absence of any structural brain abnormalities, EEG suggested injury-induced functional disorders.
Groppa et al. (2013)	13 healthy	Coherence	Probabilistic tractography	FA values in and around thalamus contralateral to the TMS pulse as well as in transcallosal fiber tracts showed a positive correlation with changes in alpha coherence bilateral, central electrodes.
Inoue (2012)	2 unilateral asterixis	Short-time Fourier transform	Probabilistic tractography	Thalamic lesion involving the ventral lateral nucleus seemingly correlated with abnormally synchronized activity of the beta band in the sensorimotor cortex
Kim et al. (2017)	10 epilepsy	sLORETA	Probabilistic tractography	EEG identified the locations with maximal cortical recruitment responses in left cingulate gyrus and right medial frontal cortex. DTI revealed fiber tracts between the centro thalamic nucleus and source-localized regions.
Nakamura-Palacios et al. (2016)	14/8 OA<br>3/6 OCC<br>7/7 ICC<br>(sham/real)	ERP based P300	Deterministic tractography	Ventral medial pre-frontal cortex appeared to be the brain region with the highest drug-related activity change within the P300 segment after treatment. DTI showed increased FA values of fiber tracts between pre-frontal cortex and nucleus accumbens in the left hemisphere after treatment.

Abbrev: Spectrum-weighted adaptive transfer function (swADTF), outpatient crack-cocaine addicts (OCC), inpatient crack-cocaine addicts (ICC), outpatient alcohol addicts (OA), standardized low-resolutions brain electromagnetic tomography (sLORETA).

S2 Primarily clinical studies

For the sake of completeness, we also list the studies that we excluded since they employed the modalities for their isolated diagnostic value. They fall in two categories, namely studies on epilepsy (Table S2) and studies on Creutzfeldt-Jakob disease (Table S3). Both cases contain a plenitude of case studies though several exception come with a substantial sample size.

Table S2: Studies on epilepsy.

	# patients (+controls)		# patients (+controls)		# patients (+controls)
Arfanakis et al. (2002)	15 (+15)	Hammen et al. (2020)	10 (+10)	Natsume et al. (2007)	12 (+13)
Assaf et al. (2003)	12 (+14)	Hong et al. (2004)	1	Nilsson et al. (2008)	8 (+10)
Belcastro et al. (2011)	1	Hunold et al. (2014)	1	(Nooraine et al. 2014)	7
Bello-Espinosa (2015)	1	Hutchinson et al. (2010)	19 (+11)	O'Brien et al. (2007)	10 (+10)
Chandra et al. (2006)	15	Katramados et al. (2009)	36	Parmar et al. (2006)	12
Chatzikonstantinou et al. (2011)	54	Kiehna et al. (2016)	8	Pauletto et al. (2021)	1
(Cho, Yi, and Motamedi 2010)	1	Kimiwada et al. (2006)	14	Raghavendra et al. (2007)	4
Cianfoni et al. (2013)	27	Knake et al. (2009)	12 (+12)	Rennebaum (2016)	69
Ciumas et al. (2014)	25	Kokkinos et al. (2011)	1	Riley et al. (2010)	12 (+10)
Concha et al. (2012)	30 (+21)	Konermann et al. (2003)	12 (+10)	Rugg-Gunn et al. (2001)	40 (+30)
Crocker et al. (2021)	11	Krakow et al. (1999)	1	Schmidt et al. (2021)	51
Di Bonaventura et al. (2009)	10	Kuster et al. (2007)	1	Senn et al. (2003)	1
Diehl et al. (2005)	18 (+27)	Lascano et al. (2013)	1	Shigeto (2005)	2
Diehl et al. (2001)	9	Lee et al. (2015)	16 (+16)	Szabo et al. (2005)	10
Evstigneev et al. (2013)	46 (+10)	Li (2010)	18 (+12)	Toledo et al. (2008)	8
Fallahi et al. (2021)	35	(Matsumoto et al. 2020)	1	Widjaja et al. (2014)	43 (+44)
Flacke et al. (2000)	1	McGill et al. (2014)	27 (+27)	Zhang et al. (2016)	18 (+18)
Gao et al. (2012)	8 (+8)	Mohamed et al. (2007)	1	Zhang (2013)	15 (+18)
Nagai et al. (2016)	1	Nagai et al. (2016)	1		

Table S3: Studies on Creutzfeldt-Jakob disease.

	# patients
Amano et al. (2015)	1
Appel et al. (2012)	13
Bavis et al. (2003)	1
Bekiesinska-Figatowska et al. (2012)	2
Bozluolcay et al. (2014)	20
Deguchi et al. (2012)	1
(Geschwind et al. 2009)	1
Hamaguchi et al. (2005)	8
Kandiah (2008)	14
Lyytinen et al. (2010)	1
Machado et al. (2009)	1
Mahale et al. (2015)	8
Park et al. (2016)	36
Riva-Amarante et al. (2011)	7
Sarraf, Ghajarzadeh, and Salarian (2014)	1
Shi et al. (2014)	1
Shi et al. (2013)	1
(Shiga 2004)	36
Valverde (2011)	1
Wang et al. (2013)	48
Zhao et al. (2013)	57

Supplementary references

Amano, Y., N. Kimura, T. Hanaoka, Y. Aso, T. Hirano, H. Murai, K. Satoh, and E. Matsubara. 2015. "Creutzfeldt-Jakob Disease with a prion protein gene codon 180 mutation presenting asymmetric cortical high-intensity on magnetic resonance imaging." *Prion* 9 (1):29-33. doi: 10.1080/19336896.2015.1017703.

Amico, E. U. G., O. Bodart, M. Rosanova, O. Gosseries, L. Heine, P. Van Mierlo, C. Martial, M. Massimini, D. P. P. Marinazzo, and S. Laureys. 2017. "Tracking dynamic interactions between structural and functional connectivity : a TMS/EEG-dMRI study." *Brain Connectivity*.

Amico, E., P. Van Mierlo, D. Marinazzo, and S. Laureys. 2015. "Investigating dynamical information transfer in the brain following a TMS pulse: Insights from structural architecture." *Conference proceedings - IEEE engineering in medicine and biology society* 2015:5396-9. doi: 10.1109/EMBC.2015.7319611.

Appel, S. A., J. Chapman, I. Prohovnik, C. Hoffman, O. S. Cohen, and I. Blatt. 2012. "The EEG in E200K familial CJD: relation to MRI patterns." *J Neurol* 259 (3):491-6. doi: 10.1007/s00415-011-6208-5.

Arfanakis, K., B. P. Hermann, B. P. Rogers, J. D. Carew, M. Seidenberg, and M. E. Meyerand. 2002. "Diffusion tensor MRI in temporal lobe epilepsy." *Magn Reson Imaging* 20 (7):511-9.

Assaf, B. A., F. B. Mohamed, K. J. Abou-Khaled, J. M. Williams, M. S. Yazeji, J. Haselgrove, and S. H. Faro. 2003. "Diffusion tensor imaging of the hippocampal formation in temporal lobe epilepsy." *AJNR Am J Neuroradiol* 24 (9):1857-62.

Bashir, S., M. Vernet, W. K. Yoo, I. Mizrahi, H. Theoret, and A. Pascual-Leone. 2012. "Changes in cortical plasticity after mild traumatic brain injury." *Restor Neurol Neurosci* 30 (4):277-82. doi: 10.3233/RNN-2012-110207.

Bavis, J., P. Reynolds, C. Tegeler, and P. Clark. 2003. "Asymmetric neuroimaging in Creutzfeldt-Jakob disease: a ruse." *J Neuroimaging* 13 (4):376-9.

Bekiesinska-Figatowska, M., A. Kuczynska-Zardzewialy, B. Pomianowska, K. Kajdana, G. M. Szpak, B. Iwanowska, and J. Madzik. 2012. "The value of magnetic resonance imaging in the early diagnosis of Creutzfeldt-Jakob disease - own experience." *Pol J Radiol* 77 (1):63-7.

Belcastro, V., P. Striano, L. Pierguidi, P. Calabresi, and N. Tambasco. 2011. "Ictal epileptic headache mimicking status migrainosus: EEG and DWI-MRI findings." *Headache* 51 (1):160-2. doi: 10.1111/j.1526-4610.2010.01709.x.

Bello-Espinosa, L. E. 2015. "Infraslow status epilepticus: A new form of subclinical status epilepticus recorded in a child with Sturge-Weber syndrome." *Epilepsy Behav* 49:193-7. doi: 10.1016/j.yebeh.2015.04.031.

Bortoletto, M., L. Bonzano, A. Zazio, C. Ferrari, L. Pedullà, R. Gasparotti, C. Miniussi, and M. Bove. 2021. "Asymmetric transcallosal conduction delay leads to finer bimanual coordination." *Brain Stimulation* 14 (2):379-388. doi: 10.1016/j.brs.2021.02.002.

Bozluolcay, M., A. D. Elmali, S. F. Menku, B. Zeydan, G. Benbir, S. Delil, and N. Yeni. 2014. "Magnetic resonance imaging findings in probable Creutzfeld-Jacob disease: comparison with electroencephalography and cerebrospinal fluid characteristics." *Acta Radiol Short Rep* 3 (10):2047981614552218. doi: 10.1177/2047981614552218.

Chandra, P. S., N. Salamon, J. Huang, J. Y. Wu, S. Koh, H. V. Vinters, and G. W. Mathern. 2006. "FDG-PET/MRI coregistration and diffusion-tensor imaging distinguish epileptogenic tubers and cortex in patients with tuberous sclerosis complex: a preliminary report." *Epilepsia* 47 (9):1543-9. doi: 10.1111/j.1528-1167.2006.00627.x.

Chatzikonstantinou, A., A. Gass, A. Forster, M. G. Hennerici, and K. Szabo. 2011. "Features of acute DWI abnormalities related to status epilepticus." *Epilepsy Res* 97 (1-2):45-51. doi: 10.1016/j.eplepsyres.2011.07.002.

Cho, Y. W., S. D. Yi, and G. K. Motamedi. 2010. "Frontal lobe epilepsy may present as myoclonic seizures." *Epilepsy Behavior* 17 (4):561-4. doi: 10.1016/j.yebeh.2010.01.022.

Cianfoni, A., M. Caulo, A. Cerase, G. Della Marca, C. Falcone, G. M. Di Lella, S. Gaudino, J. Edwards, and C. Colosimo. 2013. "Seizure-induced brain lesions: a wide spectrum of variably reversible MRI abnormalities." *Eur J Radiol* 82 (11):1964-72. doi: 10.1016/j.ejrad.2013.05.020.

Ciumas, C., M. Saignavongs, F. Ilski, V. Herbillon, A. Laurent, A. Lothe, R. A. Heckemann, J. de Bellescize, E. Panagiotakaki, S. Hannoun, D. S. Marinier, A. Montavont, K. Ostrowsky-Coste, N. Bedoin, and P. Ryvlin. 2014. "White matter development in children with benign childhood epilepsy with centro-temporal spikes." *Brain* 137 (Pt 4):1095-106. doi: 10.1093/brain/awu039.

Concha, L., H. Kim, A. Bernasconi, B. C. Bernhardt, and N. Bernasconi. 2012. "Spatial patterns of water diffusion along white matter tracts in temporal lobe epilepsy." *Neurology* 79 (5):455-62. doi: 10.1212/WNL.0b013e31826170b6.

Crocker, B., L. Ostrowski, Z. M. Williams, D. D. Dougherty, E. N. Eskandar, A. S. Widge, C. J. Chu, S. S. Cash, and A. C. Paulk. 2021. "Local and distant responses to single pulse electrical stimulation reflect different forms of connectivity." *NeuroImage* 237:118094. doi: 10.1016/j.neuroimage.2021.118094.

Deguchi, K., M. Takamiya, S. Deguchi, N. Morimoto, T. Kurata, Y. Ikeda, and K. Abe. 2012. "Spreading brain lesions in a familial Creutzfeldt-Jakob disease with V180I mutation over 4 years." *BMC Neurol* 12:144. doi: 10.1186/1471-2377-12-144.

Di Bonaventura, C., F. Bonini, J. Fattouch, F. Mari, S. Petrucci, M. Carni, E. Tinelli, P. Pantano, S. Bastianello, B. Maraviglia, M. Manfredi, M. Prencipe, and A. T. Giallonardo. 2009. "Diffusion-weighted magnetic resonance imaging in patients with partial status epilepticus." *Epilepsia* 50 Suppl 1:45-52. doi: 10.1111/j.1528-1167.2008.01970.x.

Diehl, B., I. Najm, P. Ruggieri, J. Tkach, A. Mohamed, H. Morris, E. Wyllie, E. Fisher, J. Duda, M. Lieber, W. Bingaman, and H. O. Luders. 2001. "Postictal diffusion-weighted imaging for the localization of focal epileptic areas in temporal lobe epilepsy." *Epilepsia* 42 (1):21-8.

Diehl, B., M. R. Symms, P. A. Boulby, T. Salmenpera, C. A. Wheeler-Kingshott, G. J. Barker, and J. S. Duncan. 2005. "Postictal diffusion tensor imaging." *Epilepsy Res* 65 (3):137-46. doi: 10.1016/j.eplepsyres.2005.05.007.

Evstigneev, V. V., V. V. Kistsen, I. V. Bulaev, and R. A. Sakovich. 2013. "The effect of structural white matter abnormalities on the clinical course of epilepsy." *Adv Clin Exp Med* 22 (4):529-37.

Fallahi, A., M. Pooyan, J. M. Habibabadi, and M. R. Nazem-Zadeh. 2021. "Comparison of multimodal findings on epileptogenic side in temporal lobe

epilepsy using self-organizing maps." *Magnetic Resonance Materials in Physics, Biology and Medicine*. doi: 10.1007/s10334-021-00948-7.

Flacke, S., U. Wullner, E. Keller, F. Hamzei, and H. Urbach. 2000. "Reversible changes in echo planar perfusion- and diffusion-weighted MRI in status epilepticus." *Neuroradiology* 42 (2):92-5.

Gao, Y., Y. Zhang, C. S. Wong, P. M. Wu, Z. Zhang, J. Gao, D. Qiu, and B. Huang. 2012. "Diffusion abnormalities in temporal lobes of children with temporal lobe epilepsy: a preliminary diffusional kurtosis imaging study and comparison with diffusion tensor imaging." *NMR Biomed* 25 (12):1369-77. doi: 10.1002/nbm.2809.

Geschwind, M. D., C. A. Potter, M. Sattavat, P. A. Garcia, H. J. Rosen, B. L. Miller, and S. J. DeArmond. 2009. "Correlating DWI MRI with pathologic and other features of Jakob-Creutzfeldt disease." *Alzheimer Dis Assoc Disord* 23 (1):82-87.

Ghazizadeh, A., F. Ambroggi, N. Odean, and H. L. Fields. 2012. "Prefrontal cortex mediates extinction of responding by two distinct neural mechanisms in accumbens shell." *The Journal of Neuroscience* 32 (2):726-37. doi: 10.1523/JNEUROSCI.3891-11.2012.

Groppa, S., M. Muthuraman, B. Otto, G. Deuschl, H. R. Siebner, and J. Raethjen. 2013. "Subcortical substrates of TMS induced modulation of the cortico-cortical connectivity." *Brain Stimulation* 6 (2):138-46. doi: 10.1016/j.brs.2012.03.014.

Hamaguchi, T., T. Kitamoto, T. Sato, H. Mizusawa, Y. Nakamura, M. Noguchi, Y. Furukawa, C. Ishida, I. Kuji, K. Mitani, S. Murayama, T. Kohriyama, S. Katayama, M. Yamashita, T. Yamamoto, F. Udaka, A. Kawakami, Y. Ihara, T. Nishinaka, S. Kuroda, N. Suzuki, Y. Shiga, H. Arai, M. Maruyama, and M. Yamada. 2005. "Clinical diagnosis of MM2-type sporadic Creutzfeldt-Jakob disease." *Neurology* 64 (4):643-8. doi: 10.1212/01.WNL.0000151847.57956.FA.

Hammen, T., M. Reisert, W. Juschkat, K. Egger, H. Urbach, J. Zentner, J. Beck, H. Hamer, B. J. Steinhoff, C. Baumgartner, A. Schulze-Bonhage, and B. Puhahn-Schmeiser. 2020. "Alterations of intracerebral connectivity in epilepsy patients with secondary bilateral synchrony." *Epilepsy Research* 166:106402. doi: 10.1016/j.eplepsyres.2020.106402.

Hong, K. S., Y. J. Cho, S. K. Lee, S. W. Jeong, W. K. Kim, and E. J. Oh. 2004. "Diffusion changes suggesting predominant vasogenic oedema during partial status epilepticus." *Seizure* 13 (5):317-21. doi: 10.1016/j.seizure.2003.08.004.

Hunold, A., J. Haueisen, B. Ahtam, C. Doshi, C. Harini, S. Camposano, S. K. Warfield, P. E. Grant, Y. Okada, and C. Papadelis. 2014. "Localization of the epileptogenic foci in tuberous sclerosis complex: a pediatric case report." *Front Hum Neurosci* 8:175. doi: 10.3389/fnhum.2014.00175.

Hutchinson, E., D. Pulsipher, K. Dabbs, A. Myers y Gutierrez, R. Sheth, J. Jones, M. Seidenberg, E. Meyerand, and B. Hermann. 2010. "Children with new-onset epilepsy exhibit diffusion abnormalities in cerebral white matter in the absence of volumetric differences." *Epilepsy Res* 88 (2-3):208-14. doi: 10.1016/j.eplepsyres.2009.11.011.

Inoue, M; Kojima, Y; Mima, T; Sawamoto, N; Matsuhashi, M; Fumuro, T; Kinboshi, M; Koganemaru, S; Kanda, M; Shibasaki, H. 2012. "Pathophysiology of unilateral asterixis due to thalamic lesion." *Clin Neurophysiol* 123 (9):1858-64.

Kandiah, N.; Tan, K.; Pan, A.B.; Au, W.L.; Venketasubramanian, N.; Tchoyoson Lim, C.C.; Tan, N.C.;. 2008. "Creutzfeldt-Jakob disease: Which diffusion-weighted imaging abnormality is associated with periodic EEG complexes?" *Journal of neurology* 255 (9):1411-4.

Katramados, A. M., D. Burdette, S. C. Patel, L. R. Schultz, S. Gaddam, and P. D. Mitsias. 2009. "Periictal diffusion abnormalities of the thalamus in partial status epilepticus." *Epilepsia* 50 (2):265-75. doi: 10.1111/j.1528-1167.2008.01736.x.

Kiehna, E. N., E. Widjaja, S. Holowka, O. Carter Snead, 3rd, J. Drake, S. K. Weiss, A. Ochi, E. M. Thompson, C. Go, H. Otsubo, E. J. Donner, and J. T. Rutka. 2016. "Utility of diffusion tensor imaging studies linked to neuronavigation and other modalities in repeat hemispherotomy for intractable epilepsy." *J Neurosurg Pediatr* 17 (4):483-90. doi: 10.3171/2015.7.PEDS15101.

Kim, S. H., S. C. Lim, D. W. Yang, J. H. Cho, B. C. Son, J. Kim, S. B. Hong, and Y. M. Shon. 2017. "Thalamo-cortical network underlying deep brain stimulation of centromedian thalamic nuclei in intractable epilepsy: a multimodal imaging analysis." *Neuropsychiatric Disease and Treatment* 13:2607-2619. doi: 10.2147/NDT.S148617.

Kimiwada, T., C. Juhasz, M. Makki, O. Muzik, D. C. Chugani, E. Asano, and H. T. Chugani. 2006. "Hippocampal and thalamic diffusion abnormalities in children with temporal lobe epilepsy." *Epilepsia* 47 (1):167-75. doi: 10.1111/j.1528-1167.2006.00383.x.

Knake, S., D. H. Salat, E. Halgren, M. A. Halko, D. N. Greve, and P. E. Grant. 2009. "Changes in white matter microstructure in patients with TLE and hippocampal sclerosis." *Epileptic Disord* 11 (3):244-50. doi: 10.1684/epd.2009.0272.

Kokkinos, V., K. Garganis, K. Kontogiannis, and B. Zountsas. 2011. "Hemispherotomy or lobectomy? The role of presurgical neuroimaging in a young case of a large porencephalic cyst with intractable epilepsy." *Pediatr Neurosurg* 47 (3):204-9. doi: 10.1159/000330546.

Konermann, S., S. Marks, T. Ludwig, J. Weber, A. de Greiff, A. Dorfler, G. Leonhardt, H. Wiedemayer, H. C. Diener, and A. Hufnagel. 2003. "Presurgical evaluation of epilepsy by brain diffusion: MR-detected effects of flumazenil on the epileptogenic focus." *Epilepsia* 44 (3):399-407.

Krakow, K., U. C. Wieshmann, F. G. Woermann, M. R. Symms, M. A. McLean, L. Lemieux, P. J. Allen, G. J. Barker, D. R. Fish, and J. S. Duncan. 1999. "Multimodal MR imaging: functional, diffusion tensor, and chemical shift imaging in a patient with localization-related epilepsy." *Epilepsia* 40 (10):1459-62.

Kuster, G. W., P. Braga-Neto, D. Santos-Neto, M. T. Garcia Santana, A. C. Maia Jr, and O. G. Povoas Barsottini. 2007. "Hippocampal sclerosis and status epilepticus: cause or consequence? A MRI study." *Arq Neuropsiquiatr* 65 (4B):1101-4.

Lascano, A. M., A. Lemkaddem, C. Granziera, C. M. Korff, C. Boex, B. Jenny, T. Schmitt-Mechelke, J. P. Thiran, V. Garibotto, M. I. Vargas, K. Schaller, M. Seeck, and S. Vulliemoz. 2013. "Tracking the source of cerebellar epilepsy: hemifacial seizures associated with cerebellar cortical dysplasia." *Epilepsy Res* 105 (1-2):245-9. doi: 10.1016/j.eplepsyres.2012.12.010.

Lee, S., J. B. Allendorfer, T. E. Gaston, J. C. Griffis, K. A. Hernando, R. C. Knowlton, J. P. Szaflarski, and L. W. Ver Hoef. 2015. "White matter diffusion abnormalities in patients with psychogenic non-epileptic seizures." *Brain Res* 1620:169-76. doi: 10.1016/j.brainres.2015.04.050.

Levy-Lamdan, O., N. Zifman, E. Sasson, S. Efrati, D. C. Hack, D. Tanne, I. Dolev, and H. Fogel. 2020. "Evaluation of White Matter Integrity Utilizing the DELPHI (TMS-EEG) System." *Frontiers in Neuroscience* 14:589107. doi: 10.3389/fnins.2020.589107.

Li, H.; Xue, Z.; Dulay, M. F.; Verma, A.; Wong, S.; Karmonik, C.; Grossman, R.; Wong, S. T. 2010. "Distinguishing Left or Right Temporal Lobe Epilepsy from Controls Using Fractional Anisotropy Asymmetry Analysis." *MEDICAL IMAGING AND AUGMENTED REALITY*:219-227.

Lyytinen, J., T. Sairanen, L. Valanne, T. Salmi, A. Paetau, and E. Pekkonen. 2010. "Progressive Stroke-Like Symptoms in a Patient with Sporadic Creutzfeldt-Jakob Disease." *Case Rep Neurol* 2 (1):12-18. doi: 10.1159/000289177.

Machado, A., M. Ribeiro, M. Rodrigues, C. Ferreira, I. Baldeiras, M. H. Ribeiro, I. Santana, R. Almeida, L. Castro, and S. Carpenter. 2009. "Sporadic Creutzfeldt-Jakob disease causing a 2-years slowly progressive isolated dementia." *Behav Neurol* 21 (3):175-9. doi: 10.3233/BEN-2009-0238.

Mahale, R. R., M. Javali, A. Mehta, S. Sharma, P. Acharya, and R. Srinivasa. 2015. "A study of clinical profile, radiological and electroencephalographic characteristics of suspected Creutzfeldt-Jakob disease in a tertiary care centre in South India." *J Neurosci Rural Pract* 6 (1):39-50. doi: 10.4103/0976-3147.143189.

Matsumoto, Y., Y. Akamatsu, Y. Ogasawara, K. Aso, S. Oshida, and H. Kashimura. 2020. "A case of paroxysmal homonymous hemianopsia: Uncommon presentation of nonconvulsive status epilepticus." *Radiol Case Rep* 15 (6):668-671. doi: 10.1016/j.radcr.2020.03.005.

McGill, M. L., O. Devinsky, X. Wang, B. T. Quinn, H. Pardoe, C. Carlson, T. Butler, R. Kuzniecky, and T. Thesen. 2014. "Functional neuroimaging abnormalities in idiopathic generalized epilepsy." *Neuroimage Clin* 6:455-62. doi: 10.1016/j.nicl.2014.10.008.

Mohamed, I. S., H. Otsubo, M. Shroff, E. Donner, J. Drake, and O. C. Snead, 3rd. 2007. "Magnetoencephalography and diffusion tensor imaging in gelastic seizures secondary to a cingulate gyrus lesion." *Clin Neurol Neurosurg* 109 (2):182-7. doi: 10.1016/j.clineuro.2006.06.005.

Momi, D., R. A. Ozdemir, E. Tadayon, P. Boucher, A. Di Domenico, M. Fasolo, M. M. Shafi, A. Pascual-Leone, and E. Santarnecchi. 2021. "Perturbation of resting-state network nodes preferentially propagates to structurally rather than functionally connected regions." *Scientific Reports* 11 (1):12458. doi: 10.1038/s41598-021-90663-z.

Momi, D., R. A. Ozdemir, E. Tadayon, P. Boucher, M. M. Shafi, A. Pascual-Leone, and E. Santarnecchi. 2021. "Network-level macroscale structural connectivity predicts propagation of transcranial magnetic stimulation." *NeuroImage* 229:117698. doi: 10.1016/j.neuroimage.2020.117698.

Nagai, Y., A. Fujimoto, T. Okanishi, H. Motoi, S. Kanai, T. Yokota, H. Enoki, M. Nishimura, and T. Yamamoto. 2016. "Successful hemispherotomy for a patient with intractable epilepsy secondary to bilateral congenital brain malformation with lateralized pyramidal tract of diffusion tensor

image tractography." *Epilepsy Behav Case Rep* 6:30-2. doi: 10.1016/j.ebcr.2016.01.005.

Nakamura-Palacios, E. M. 2011. "Working memory and prefrontal cortex and their relation with the brain reward system and drug addiction." *Working Memory: Capacity, Developments and Improvement Techniques*:109-139.

Nakamura-Palacios, E. M., I. B. C. Lopes, R. A. Souza, J. Klauss, E. K. Batista, C. L. Conti, J. A. Moscon, and R. S. M. de Souza. 2016. "Ventral medial prefrontal cortex (vmPFC) as a target of the dorsolateral prefrontal modulation by transcranial direct current stimulation (tDCS) in drug addiction." *Journal of Neural Transmission* 123 (10):1179-1194. doi: 10.1007/s00702-016-1559-9.

Natsume, J., N. Bernasconi, M. Miyauchi, M. Naiki, T. Yokotsuka, A. Sofue, and A. Bernasconi. 2007. "Hippocampal volumes and diffusion-weighted image findings in children with prolonged febrile seizures." *Acta Neurol Scand* 115 (4 Suppl):25-8. doi: 10.1111/j.1600-0404.2007.00806.x.

Nestler, E. J. 2004. "Molecular mechanisms of drug addiction." *Neuropharmacology* 47 (1):24-32. doi: 10.1016/j.neuropharm.2004.06.031.

Nilsson, D., C. Go, J. T. Rutka, B. Rydenhag, D. J. Mabbott, O. C. Snead, 3rd, C. R. Raybaud, and E. Widjaja. 2008. "Bilateral diffusion tensor abnormalities of temporal lobe and cingulate gyrus white matter in children with temporal lobe epilepsy." *Epilepsy Res* 81 (2-3):128-35. doi: 10.1016/j.eplepsyres.2008.05.002.

Nooraine, J., Shiva K. R, R. B. Iyer, R. M. Rao, and S. Raghavendra. 2014. "Posterior quadrant disconnection for refractory epilepsy: A case series." *Annals of Indian Academy of Neurology* 17 (4):392-397. doi: 10.4103/0972-2327.144006.

O'Brien, T. J., E. P. David, C. J. Kilpatrick, P. Desmond, and B. Tress. 2007. "Contrast-enhanced perfusion and diffusion MRI accurately lateralize temporal lobe epilepsy: a pilot study." *J Clin Neurosci* 14 (9):841-9. doi: 10.1016/j.jocn.2006.07.003.

Park, S. Y., M. J. Wang, J. W. Jang, Y. H. Park, J. S. Lim, Y. C. Youn, J. Kim, and S. Kim. 2016. "The Clinical Stages of Sporadic Creutzfeldt-Jakob Disease with Met/Met Genotype in Korean Patients." *Eur Neurol* 75 (5-6):213-22. doi: 10.1159/000445768.

Parmar, H., S. H. Lim, N. C. Tan, and C. C. Lim. 2006. "Acute symptomatic seizures and hippocampus damage: DWI and MRS findings." *Neurology* 66 (11):1732-5. doi: 10.1212/01.wnl.0000218207.18707.f4.

Pauletto, G., I. Guarracino, A. Nilo, T. Ius, M. Maieron, L. Verriello, M. Skrap, G. L. Gigli, and B. Tomasino. 2021. "What's behind drawing for an artist with left temporal lobe epilepsy? A multimodal neurophysiological study." *Epilepsy & Behavior Reports* 16:100418. doi: 10.1016/j.ebr.2020.100418.

Pinter, D., S. E. Kober, V. Fruhwirth, L. Berger, A. Damulina, M. Khalil, C. Neuper, G. Wood, and C. Enzinger. 2021. "MRI correlates of cognitive improvement after home-based EEG neurofeedback training in patients with multiple sclerosis: a pilot study." *Journal of Neurology*. doi: 10.1007/s00415-021-10530-9.

Raghavendra, S., R. Ashalatha, S. V. Thomas, and C. Kesavadas. 2007. "Focal neuronal loss, reversible subcortical focal T2 hypointensity in seizures with a nonketotic hyperglycemic hyperosmolar state." *Neuroradiology* 49 (4):299-305. doi: 10.1007/s00234-006-0189-6.

Rennebaum, F; Kassubek, J; Pinkhardt, E; Hübers, A; Ludolph, AC; Schocke, M; Fauser, S. 2016. "Status epilepticus: Clinical characteristics and EEG patterns associatedwith and without MRI diffusion restriction in 69 patients." *Epilepsy Res* 120 (2):55-64.

Riley, J. D., D. L. Franklin, V. Choi, R. C. Kim, D. K. Binder, S. C. Cramer, and J. J. Lin. 2010. "Altered white matter integrity in temporal lobe epilepsy: association with cognitive and clinical profiles." *Epilepsia* 51 (4):536-45. doi: 10.1111/j.1528-1167.2009.02508.x.

Riva-Amarante, E., A. Jimenez-Huete, R. Toledano, M. Calero, J. Alvarez-Linera, J. Escribano, M. J. Sanchez Migallon, and O. Franch. 2011. "Usefulness of high b-value diffusion-weighted MRI in the diagnosis of Creutzfeldt-Jakob disease." *Neurologia* 26 (6):331-6. doi: 10.1016/j.nrl.2010.12.003.

Rudorf, S., and T. A. Hare. 2014. "Interactions between dorsolateral and ventromedial prefrontal cortex underlie context-dependent stimulus valuation in goal-directed choice." *The Journal of neuroscience* 34 (48):15988-15996. doi: 10.1523/JNEUROSCI.3192-14.2014.

Rugg-Gunn, F. J., S. H. Eriksson, M. R. Symms, G. J. Barker, and J. S. Duncan. 2001. "Diffusion tensor imaging of cryptogenic and acquired partial epilepsies." *Brain* 124 (Pt 3):627-36.

Sarraf, P., M. Ghajarzadeh, and B. Salarian. 2014. "Creutzfeldt-Jacob disease: a case report." *Acta Med Iran* 52 (6):488-9.

Schmidt, M. H., C. E. Crocker, M. Abdolell, M. S. Ghuman, and B. Pohlmann-Eden. 2021. "Toward individualized prediction of seizure recurrence: Hippocampal neuroimaging features in a cohort of patients from a first seizure clinic." *Epilepsy & Behavior* 122:108118. doi: 10.1016/j.yebeh.2021.108118.

Senn, P., K. O. Lovblad, D. Zutter, C. Bassetti, O. Zeller, F. Donati, and G. Schroth. 2003. "Changes on diffusion-weighted MRI with focal motor status epilepticus: case report." *Neuroradiology* 45 (4):246-9. doi: 10.1007/s00234-002-0850-7.

Shi, Q., C. Chen, X. J. Wang, W. Zhou, J. C. Wang, B. Y. Zhang, C. Gao, C. Gao, J. Han, and X. P. Dong. 2013. "Rare V203I mutation in the PRNP gene of a Chinese patient with Creutzfeldt-Jakob disease." *Prion* 7 (3):259-62. doi: 10.4161/pri.24674.

Shi, Q., X. J. Shen, W. Zhou, K. Xiao, X. M. Zhang, B. Y. Zhang, and X. P. Dong. 2014. "Rare V180I mutation in PRNP gene of a Chinese patient with Creutzfeldt-Jakob disease." *Prion* 8 (6):411-4. doi: 10.4161/19336896.2014.967040.

Shiga, Y.; Miyazawa, K.; Sato, S.; Fukushima, R.; Shibuya, S.; Sato, Y.; Konno, H.; Doh-ura, K.; Mugikura, S.; Tamura, H.; Higano, S.; Takahashi, S.; Itoyama, Y.;. 2004. "Diffusion-weighted MRI abnormalities as an early diagnostic marker for Creutzfeldt-Jakob disease." *Neurology* 63 (3):443-9.

Shigeto, H; Uehara, T; Uchida, K; Nomura, T; Taniwaki, T; Kira, JI. 2005. "Thalamic involvement of status epilepticus: diffusion-weighted image of MRI in two cases of status epilepticus." *International Congress Series* 1278:193–196.

Szabo, K., A. Poepel, B. Pohlmann-Eden, J. Hirsch, T. Back, O. Sedlaczek, M. Hennerici, and A. Gass. 2005. "Diffusion-weighted and perfusion MRI demonstrates parenchymal changes in complex partial status epilepticus." *Brain* 128 (Pt 6):1369-76. doi: 10.1093/brain/awh454.

Toledo, M., J. Munuera, M. Sueiras, R. Rovira, J. Alvarez-Sabin, and A. Rovira. 2008. "MRI findings in aphasic status epilepticus." *Epilepsia* 49 (8):1465-9. doi: 10.1111/j.1528-1167.2008.01620.x.

Valverde, A.H.; Costa, S.; Timoteo, Â.; Ginestal, R.; Pimentel, J.;. 2011. "Rapidly Progressive Corticobasal Degeneration Syndrome." *Case reports in neurology* 3 (2):185–190.

Wang, L. H., R. C. Bucelli, E. Patrick, D. Rajderkar, E. Alvarez Iii, M. M. Lim, G. Debruin, V. Sharma, S. Dahiya, R. E. Schmidt, T. S. Benzinger, B. A. Ward, and B. M. Ances. 2013. "Role of magnetic resonance imaging, cerebrospinal fluid, and electroencephalogram in diagnosis of sporadic Creutzfeldt-Jakob disease." *J Neurol* 260 (2):498-506. doi: 10.1007/s00415-012-6664-6.

Weichart, E. R., P. B. Sederberg, F. Sammartino, V. Krishna, J. D. Corrigan, and A. R. Rezai. 2020. "Cognitive Task Performance During Titration Predicts Deep Brain Stimulation Treatment Efficacy: Evidence From a Case Study." *Frontiers in Psychiatry* 11:30. doi: 10.3389/fpsyt.2020.00030.

Whitmer, D., C. de Solages, B. Hill, H. Yu, J. M. Henderson, and H. Bronte-Stewart. 2012. "High frequency deep brain stimulation attenuates subthalamic and cortical rhythms in Parkinson's disease." *Frontiers Humman Neuroscience* 6:155. doi: 10.3389/fnhum.2012.00155.

Widjaja, E., A. Kis, C. Go, O. C. Snead, 3rd, and M. L. Smith. 2014. "Bilateral white matter abnormality in children with frontal lobe epilepsy." *Epilepsy Res* 108 (2):289-94. doi: 10.1016/j.eplepsyres.2013.12.001.

Zhang, Y; Yan, X; Gao, Y; Xu, D; Wu, J; Li, Y. 2013. "A preliminary study of epilepsy in children using diffusional kurtosis imaging." *Clin Neuroradiol* 23 (4):293-300.

Zhang, Y., Y. Gao, M. Zhou, J. Wu, C. Zee, and D. Wang. 2016. "A diffusional kurtosis imaging study of idiopathic generalized epilepsy with unilateral interictal epileptiform discharges in children." *J Neuroradiol* 43 (5):339-45. doi: 10.1016/j.neurad.2016.05.001.

Zhao, W., J. T. Zhang, X. W. Xing, D. H. Huang, C. L. Tian, W. Q. Jia, X. S. Huang, W. P. Wu, C. Q. Pu, S. Y. Lang, and S. Y. Yu. 2013. "Chinese specific characteristics of sporadic Creutzfeldt-Jakob disease: a retrospective analysis of 57 cases." *PLoS One* 8 (3):e58442. doi: 10.1371/journal.pone.0058442.

Zumsteg, D., A. M. Lozano, and R. A. Wennberg. 2006. "Rhythmic cortical EEG synchronization with low frequency stimulation of the anterior and medial thalamus for epilepsy." *Clinical Neurophysiology* 117 (10):2272-8. doi: 10.1016/j.clinph.2006.06.707.

Zumsteg, D., A. M. Lozano, H. G. Wieser, and R. A. Wennberg. 2006. "Cortical activation with deep brain stimulation of the anterior thalamus for epilepsy." *Clinical Neurophysiology* 117 (1):192-207. doi: 10.1016/j.clinph.2005.09.015.
